# Mechanistic Structure–Property Relationships in Carbon/Polymer Composites: Connectivity, Junction Resistance, and Durability

**DOI:** 10.3390/polym18101220

**Published:** 2026-05-16

**Authors:** Sachin Kumar Sharma, Reshab Pradhan, Lokesh Kumar Sharma, Yogesh Sharma, Yatendra Pal, Drago Bračun, Damjan Klobčar

**Affiliations:** 1 Surface Science and Tribology Lab, Department of Mechanical Engineering, Shiv Nadar Institution of Eminence, Gautam Buddha Nagar 201314, India; ss393@snu.edu.in (S.K.S.); rp943@snu.edu.in (R.P.); 2 Department of Physics, GLA University, Mathura 281406, India; lokesh.sharma@gla.ac.in; 3 Department of Physics & Environmental Sciences, Sharda School of Engineering & Science, Sharda University, Greater Noida 201310, India; yogesh.sharma2@sharda.ac.in; 4 Institute of Education and Research, Mangalayatan University, Aligarh 202146, India; yp.gaur257@gmail.com; 5 Faculty of Mechanical Engineering, University of Ljubljana, Aškerčeva 6, 1000 Ljubljana, Slovenia; drago.bracun@fs.uni-lj.si

**Keywords:** carbon/polymer composites, percolation and tunneling, interfacial thermal resistance, hybrid and 3D architecture, durability and recyclability

## Abstract

Carbon/polymer composites are increasingly designed as microstructure-engineered multifunctional materials that combine mechanical reinforcement with electrical/thermal transport, electromagnetic interference (EMI) shielding, and sensing. Performance is governed less by filler fraction than by the coupled control of network topology, junction resistance, and interfacial thermal boundary resistance under processing-induced shear and thermal histories. Electrical response follows percolation combined with tunneling/contact-controlled junctions, producing nonlinear σ(φ) behavior and high piezoresistive sensitivity near the percolation threshold. In contrast, thermal transport is commonly limited by Kapitza resistance and filler–filler junction resistance, restricting exploitation of the intrinsic conductivity of CNTs and graphene. Recent advances emphasize hybrid and 3D carbon architectures that densify connectivity, reduce junction losses, and enable programmable anisotropy via scalable routes such as masterbatch extrusion and additive manufacturing. However, translation remains constrained by dispersion-driven variability, transport–toughness trade-offs, and incomplete durability assessment under cycling, humidity, and reprocessing. This review consolidates mechanistic structure–processing–property relationships and provides application-driven design rules for sensors, EMI shielding, and thermal management.

## 1. Introduction

Carbon/polymer composites have transitioned from particulate-filled plastics to multifunctional systems where mechanical reinforcement, electrical/thermal transport, and electromagnetic response are governed by microstructure-controlled network formation [1,2,3]. Unlike conventional fiber-reinforced polymers dominated by load transfer, carbon-filled polymers behave as percolating heterogeneous media in which properties arise from the coupled evolution of dispersion/aggregation state, interfacial chemistry and adhesion, and conductive network topology imposed by processing shear histories [4,5,6]. Consequently, carbonaceous fillers alter stiffness and strength as well as melt rheology, interphase mobility, defect nucleation, and charge/heat transport pathways [7,8,9]. This coupling explains the large property scatter and motivates a structure–processing–property approach rather than filler loading-only interpretation. Carbon fillers span multiple length scales and dimensionalities, including carbon black (0D), CNT/CNF (1D), graphene derivatives (2D), and 3D architectures such as foams, aerogels, and vertically aligned networks [10,11,12]. Carbon black typically requires high loading to reach electrical percolation due to limited aspect ratios, often causing viscosity rise and embrittlement [13,14]. CNT/CNF networks achieve conduction at low loading and enable piezoresistive sensitivity near percolation [15,16,17], but agglomeration suppresses effective aspect ratio and degrades both reinforcement and network continuity [18,19]. Graphene derivatives provide high intrinsic in-plane transport but suffer from restacking and intersheet contact resistance that limit long-range pathways [20,21]. Therefore, hybrid and hierarchical filler designs are widely used to increase junction density and stabilize dispersion (e.g., CNT bridging between graphene platelets), reducing the effective percolation threshold at lower loadings [22,23,24].

Applications in EMI shielding, electrostatic discharge, flexible electronics, sensors, and thermal management impose the same engineering requirement: achieve high transport performance under manufacturable filler loadings while maintaining toughness, processability, and stability [25,26,27,28]. Most functional behavior emerges near or beyond the percolation threshold, where filler connectivity generates long-range networks [29]. Electrical transport is governed by direct contacts and tunneling across nanometer-scale gaps, making junction resistance dominant and conductivity highly sensitive to dispersion uniformity, alignment, and polymer dynamics that control gap stability under strain and temperature [30]. Segregated networks, hybrid fillers, and 3D frameworks reduce the percolation threshold and enable high conductivity without excessive viscosity penalties [31]. However, transport gains often compete with ductility because agglomerates and rigid networks elevate defect density and stress concentration.

Thermal transport is typically more constrained. Although CNTs and graphene have high intrinsic thermal conductivity, composites often show modest improvement because heat transfer is limited by Kapitza thermal boundary resistance and phonon scattering at filler–matrix interfaces [32,33,34]. Increasing loading rarely gives proportional gains; pathway continuity and filler–filler junction resistance govern effective conductivity [35]. Covalent functionalization improves bonding but disrupts the sp^2^ network and reduces intrinsic filler transport [36], while noncovalent approaches preserve graphitic structure but may introduce interfacial layers that increase thermal boundary resistance [37]. Continuous or quasi-continuous architectures (3D foams/aerogels, vertically aligned networks) reduce dependence on high-resistance junctions and enable anisotropic heat transport tailored to device requirements. The interface also controls mechanical response through interfacial shear transfer, matrix yielding, and crack deflection/bridging; weak interfaces promote debonding/pull-out, whereas overly strong interfaces may trigger brittle failure when agglomerates act as critical flaws [37,38]. For sensing, junction/interfacial dynamics govern reversible resistance change by tunneling gap modulation, linking sensitivity and hysteresis directly to interface design. Processing acts as a microstructure generator [39]. Melt compounding/extrusion/injection molding imposes shear that aligns fillers, fragments agglomerate, and produces anisotropic networks, while solution blending improves dispersion but limits scalability. In situ polymerization can immobilize networks yet introduce shrinkage-induced changes. Additive manufacturing (FDM, DIW) provides voxel-scale control over orientation and concentration, enabling patterned conductivity gradients, tailored EMI shielding, and programmable sensor response, although predictive processing–microstructure–property models remain limited for hybrid and anisotropic systems.

Despite extensive research, translation remains restricted by three gaps: dispersion is frequently assessed qualitatively rather than using multi-scale metrics correlated with percolation and mechanical variability; transport improvements are often reported without mechanistic decoupling of dispersion/interface/network contributions; and durability under cyclic strain, thermal aging, humidity, and electromagnetic loading is insufficiently quantified (Table 1). Sustainability and circularity (recyclable matrices, bio-derived carbons, end-of-life strategies) are also weakly integrated into functional composite design. In this context, the present review consolidates progress in carbon/polymer composites by emphasizing mechanistic relationships among architecture, interfacial engineering, percolation-dominated transport, and processing-induced anisotropy. Rather than cataloging fillers and applications, the review focuses on microstructure-governed property evolution, highlights advances in hybrid and 3D architectures and identifies design gaps that must be addressed to achieve reproducible, scalable, and sustainable multifunctional systems. To quantitatively assess the limitations of existing reviews, a comparative analysis of representative review articles published between 2018 and 2025 was conducted. It was found that only few reviews explicitly incorporate junction resistance-based transport models, while less report percolation threshold variability as a function of processing conditions. Furthermore, only few include durability data beyond 10^3^ loading cycles. These findings indicate that most existing reviews remain largely descriptive and do not integrate percolation, tunneling, and interfacial transport into a unified mechanistic framework. In contrast, the present work establishes a quantitative structure–processing–property relationship by explicitly linking network topology, junction physics, and interfacial resistance to multifunctional performance.

## 2. Carbon Architecture and Filler Design Rules for Polymer Composites

Carbon/polymer composites derive multifunctional performance from the geometry, surface state, and network-forming capability of the carbon phase. Unlike conventional particle-reinforced polymers where stiffness/strength are often interpreted through rule-of-mixtures trends, carbon-filled systems exhibit strong nonlinearity because functional properties emerge once the carbon phase forms a percolating network [40]. The composite response is therefore controlled by the coupled evolution of dispersion state, interfacial interactions, and network topology imposed by processing. This coupling explains why similar loadings can yield widely different conductivity, toughness, and reliability, motivating classification of carbon fillers by dimensionality and architecture (0D/1D/2D/3D) rather than composition alone [41]. Carbon black (0D) is the industrial baseline due to cost and maturity in elastomers and thermoplastics [42,43]. It consists of nanoscale primary particles assembled into branched aggregates/agglomerates. Reinforcement arises from hydrodynamic stiffening and polymer confinement within clusters, whereas electrical transport requires cluster–cluster connectivity [44]. Due to low effective aspect ratio, carbon black typically exhibits a higher percolation threshold than CNTs or graphene derivatives, demanding higher loading that increases viscosity and can reduce elongation and fracture resistance. Thus, carbon black alone is rarely optimal at low loading but is effective in hybrids, where it stabilizes dispersion, fills interstitial gaps, and densifies conductive pathways.

CNTs/CNFs (1D) provide high percolation efficiency because their large aspect ratio enables network formation at low volume fractions [45]. Mechanical reinforcement follows shear-lag load transfer, requiring debundling, nanotube length retention, and sufficient interfacial shear strength [46]. Agglomerates act as stress concentrators, while excessive shear shortens nanotubes, lowering effective aspect ratio and shifting the percolation threshold upward. Electrical transport combines percolation with tunneling across nanometer-scale gaps, making conductivity exponentially sensitive to junction distance distributions [47]. This also enables piezoresistive sensing but introduces variability because dispersion differences shift the network between near-percolation and well-percolated regimes. Therefore, CNT/CNF systems require strict microstructure control to ensure reproducible mechanical and functional properties [48,49]. Graphene-based fillers (2D)—GNP, GO, and rGO—provide planar architectures with large surface area and high intrinsic in-plane transport [50,51,52]. Their reinforcement involves load redistribution, crack deflection, and barrier effects, while transport depends on platelet alignment and junction quality. Restacking driven by π–π interactions limits connectivity and accessible surface area [53]. GO disperses more readily and often improves adhesion and reinforcement [54], but oxidation disrupts the sp^2^ lattice and reduces intrinsic transport; reduction partially restores conductivity but can reintroduce stacking and interfacial defects [55,56]. Hence, graphene composites are governed by a dispersion–transport trade-off [57]. In many systems, transport becomes junction-limited because platelet–platelet contact resistance dominates, particularly under alignment-induced anisotropy that improves in-plane conduction while restricting through-thickness pathways [58].

**Figure 1:**
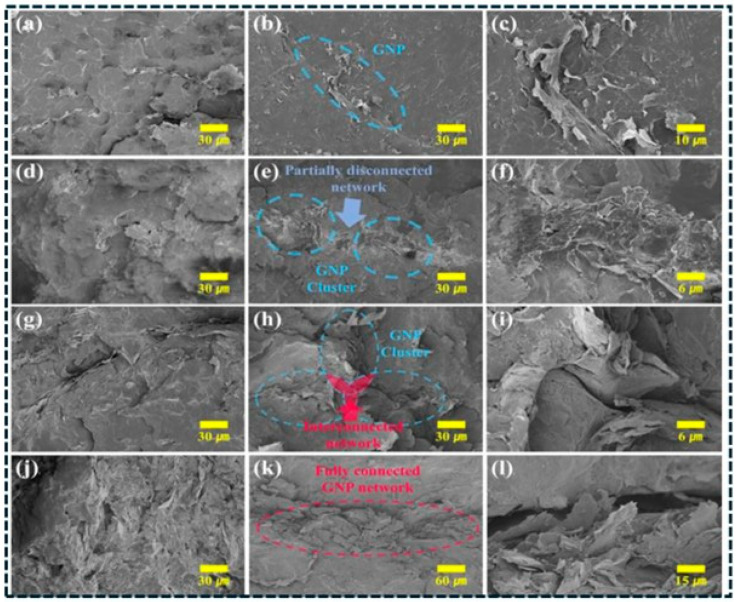
SEM micrographs (**a**–**l**) of polymer/GNP composites showing evolution from isolated platelet domains (**a**–**c**) to clustered but partially disconnected regions (**d**–**f**), progressive bridging and overlap (**g**–**i**), and a fully connected segregated GNP network (**j**–**l**) [65].

Hybrid architecture enables deliberate manipulation of topology beyond single fillers [59]. In CNT–graphene networks, synergy arises because CNTs bridge platelets and suppress restacking, while graphene provides planar highways that reduce high-resistance tube–tube junctions [60]. Hierarchical hybrids incorporating carbon black further densify networks by filling voids between high-aspect-ratio fillers, improving continuity and reducing sensitivity to local dispersion defects [61]. However, optimization often remains empirical, and predictive rules are limited regarding mixing sequence, compatibilization, and processing-induced segregation effects on network stability [62]. 3D carbon frameworks (foams, sponges, aerogels, vertically aligned networks) provide a pre-connected skeleton prior to infiltration [63], bypassing stochastic percolation and reducing tunneling dependence, enabling high conductivity at low effective filler fractions. Thermal conduction is also enhanced because continuous frameworks reduce junction resistance relative to particulate networks [64]. In these systems, performance is controlled by infiltration quality, void suppression, and framework–polymer interfacial resistance, while mechanics depends on interpenetrating-network integrity (porosity and stiffness mismatch effects). Therefore, 3D architecture enables combined EMI shielding, sensing, and thermal management but requires stringent processing control [65]. SEM evidence supports this connectivity evolution for segregated GNP networks: Figure 1a–c shows isolated regions, Figure 1d–f clustered yet partially disconnected domains, Figure 1g–i cluster bridging, and Figure 1j–l a fully connected network [65], explaining reduced percolation threshold and simultaneous transport enhancement.

Across architectures, filler selection should be guided by microstructural descriptors rather than nominal loading [66]. Effective aspect ratio, agglomerate size distribution, and spatial uniformity control percolation efficiency and mechanical scatter [67]. Connectivity metrics (contact density, cluster continuity) govern conductivity stability and sensing response. Interface engineering must consider transport: covalent functionalization improves adhesion but can degrade transport by disrupting sp^2^ networks, whereas noncovalent approaches preserve intrinsic transport but may increase boundary resistance [68]. Junction quality often dominates electrical and thermal performance and must be engineered through architecture and processing [69]. Carbon fillers should therefore be treated as network-forming architectures, not additive particles [70]. Carbon black provides robust industrial conductivity but needs higher loading; CNT/CNF networks offer low-threshold percolation but require dispersion/length control; graphene derivatives provide planar transport but remain junction/restacking-limited; hybrids provide engineered synergy; and 3D frameworks bypass percolation while shifting constraints to infiltration and interface resistance [16]. Since the polymer matrix governs dispersion kinetics and tunneling gap stability, the next section develops matrix selection rules linking polymer chemistry and rheology to network formation and multifunctional reliability. However, carbon dimensionality controls multifunctionality through connectivity and junction resistance, not filler loading alone, as shown in Table 2. 0D carbon black reinforces by aggregate-induced polymer confinement but requires high loading for percolation, increasing viscosity and lowering ductility. 1D CNT/CNF delivers the lowest percolation threshold ($p_{c}$) via high $AR_{eff}$, yet agglomeration and shear-shortening cause large scatter in σ and strength. 2D graphene composites are junction- and alignment-limited: restacking and platelet contact resistance suppress transport despite crack-deflection reinforcement. Hybrid and 3D architectures enable engineered pathways—hybrids add bridging redundancy to reduce tunneling/contact losses, while 3D frameworks bypass percolation but are limited by infiltration defects and thermal boundary resistance (TBR).

A comparative case study highlights the mechanistic advantage of hybrid architectures. In conventional CNT/epoxy systems, the percolation threshold is typically on the order of ~0.5 vol%, whereas CNT–graphene hybrid systems exhibit significantly lower thresholds (~0.1 vol%) [64]. This approximately five-fold reduction in the percolation threshold arises from CNT bridging between graphene platelets, which enhances long-range network connectivity and reduces the mean tunneling gap between conductive elements. As a result, hybrid systems achieve higher conductivity at lower filler loadings, improved junction redundancy, and reduced sensitivity to local dispersion defects [67]. This comparison demonstrates that the observed performance enhancement is governed by network topology and junction physics rather than filler composition alone. This behavior is further supported by conductivity–filler loading trends, where hybrid systems exhibit earlier percolation onset and a steeper increase in conductivity compared to single-filler systems, confirming the role of junction densification and reduced tunneling resistance [68].

## 3. Polymer Matrix Selection and Interface-Governed Multifunctionality

In carbon/polymer composites, the polymer matrix functions \ controls dispersion stability, interfacial load transfer, percolation robustness, and long-term multifunctional reliability [77]. Identical carbon architectures can exhibit markedly different mechanical and transport responses depending on matrix polarity, melt viscosity, glass transition temperature, crystallinity, and curing chemistry. Matrix selection therefore requires a mechanistic framework linking polymer thermodynamics and rheology to carbon network formation and interface-controlled transport. The matrix governs filler wetting and deagglomeration during mixing, polymer adsorption at carbon surfaces, and the evolution of tunneling gaps under strain and temperature, thereby determining both achievable performance and reproducibility. From a thermodynamic perspective, dispersion is limited by the high surface energy of carbon fillers, which promotes filler–filler attraction and agglomeration [78]. Increased matrix polarity or functional group density improve wetting and reduce interfacial tension, enabling debundling of CNTs and separation of graphene platelets [79]. However, improved dispersion does not necessarily enhance transport, because strong polymer adsorption can form electrically resistive interphases and increase thermal boundary resistance. This introduces a matrix-dependent dispersion–transport trade-off. Rheology further modulates this balance: high-viscosity melts transmit sufficient shear to disrupt agglomerates but may shorten CNTs and reduce effective aspect ratios, whereas low-viscosity melts preserve filler length but often fail to generate adequate deagglomeration stresses. Consequently, matrix viscosity must be matched to carbon architecture to preserve network connectivity while minimizing filler damage [80].

Thermoplastics dominate industrial carbon composites due to melt processability, reprocessability, and recyclability. Nonpolar thermoplastics such as polyolefins exhibit weak interactions with carbon surfaces, leading to poor wetting and unstable dispersion unless compatibilizers or functionalized fillers are employed. In these systems, the primary design objective is dispersion stabilization without excessive functionalization that disrupts the sp^2^ network. Polar thermoplastics (e.g., polyamides, polycarbonates) promote improved dispersion and stronger interfacial bonding, enhancing mechanical reinforcement at comparable loading. However, electrical transport often remains junction-limited if polymer layers increase tunneling distance or contact resistance. Semi-crystalline thermoplastics introduce additional complexity: crystallization can disrupt conductive pathways by excluding fillers from crystalline regions or form segregated networks when fillers localize at interspherulitic boundaries. Cooling rate and crystallization kinetics therefore significantly influence the percolation threshold and conductivity stability at fixed compositions. Thermoset matrices (epoxy, vinyl ester, phenolic) enable strong interfacial bonding and high stiffness, supporting structural–functional integration [81]. Carbon fillers can modify cure kinetics and crosslink density near interfaces, generating interphases with reduced polymer mobility. While this enhances stiffness and load transfer, it may suppress energy dissipation and promote brittleness if agglomerates persist. Cure shrinkage and residual stress can further perturb filler spacing, shifting the system away from optimal percolation. Electrical transport in thermosets is therefore highly sensitive to cure schedule, filler distribution prior to gelation, and post-cure thermal history. If gelation precedes network formation, percolation may not be achieved even at elevated filler loading, underscoring the need for early-stage dispersion and connectivity.

Elastomer matrices are central to flexible and wearable systems due to their ability to accommodate large strain [82]. In elastomer-based composites, electrical transport is dominated by tunneling near percolation, enabling high strain sensitivity but also introducing hysteresis and drift due to viscoelastic relaxation and irreversible microstructural rearrangement. Matrix viscoelasticity governs tunneling gap recovery and signal repeatability. Strong filler–matrix bonding enhances mechanical integrity but restricts reversible network rearrangement, reducing sensitivity, whereas weak interfaces increase sensitivity at the expense of fatigue resistance and conductivity stability. Elastomer selection must therefore be application-specific, balancing sensitivity, durability, and fatigue life rather than maximizing conductivity alone [83]. Interface engineering represents the central control point for coupling mechanical reinforcement and functional transport [84]. Load transfer requires sufficient interfacial shear strength, while fracture resistance benefits from controlled debonding and pull-out. Covalent functionalization improves adhesion and dispersion but disrupts the sp^2^ network, degrading intrinsic electrical and thermal transport. Noncovalent strategies (polymer wrapping, π–π interactions, ionic surfactants, compatibilizers) preserve electronic structure but introduce interfacial layers that increase tunneling distance and thermal boundary resistance. Interface design must therefore be evaluated using dual metrics: mechanical coupling efficiency and transport penalty. A persistent challenge is developing interfaces that maintain high shear strength while preserving sp^2^-mediated transport under cyclic strain and thermal aging. Dynamic and reversible interfacial chemistries offer promising routes by enabling mechanical integrity alongside microstructural recovery during sensing and fatigue loading.

Matrix-dependent interphase formation further influences composite behavior. Polymer chain confinement and adsorption at carbon surfaces alter local mobility, affecting rheology, glass transition behavior, creep resistance, and transport stability. In near-percolation sensing composites, the interphase governs tunneling gap stability through its control of polymer segmental motion. Temperature cycling can induce conductivity drift in matrices with high mobility, whereas rigid matrices stabilize transport but amplify interfacial thermal stresses. Moisture uptake in hydrophilic polymers can swell the matrix, increase tunneling distances, and degrade conductivity, effects often underestimated in short-term studies but dominant in long-term reliability. Processing constraints impose additional matrix selection criteria [80,81,82,83,84]. Melt-compoundable thermoplastics require processing temperatures below filler oxidation and compatibilizer degradation limits. Additive manufacturing demands rheological windows compatible with filament formation or direct-ink extrusion while maintaining dispersion and orientation stability. Thermosets used in printing or coating must retain sufficient low viscosity to enable network formation prior to gelation. Matrix selection therefore cannot be decoupled from fabrication route, as processing history directly governs anisotropy, network connectivity, and interface development. A robust design strategy jointly selects matrix and carbon architecture to target a processing window that yields reproducible dispersion and predictable percolation behavior. Accordingly, polymer matrices control carbon composite performance through dispersion thermodynamics, rheological shear transfer, interphase formation, and interface-governed transport. Thermoplastics favor scalability and recyclability but require compatibilization, thermosets offer strong load transfer with cure-sensitive network stability, and elastomers enable high sensitivity while facing fatigue and drift challenges [82]. Across all matrix classes, the central design objective is stabilizing network connectivity and load transfer while minimizing transport penalties associated with polymer adsorption and thermal boundary resistance. The following section examines manufacturing pathways as microstructure, with emphasis on scalable processing and additive manufacturing routes that enable controlled anisotropy and architected network design. Table 3 shows that matrix chemistry controls network stability by governing wetting, interphase thickness, and junction physics, which dictate contact- versus tunneling-dominated transport. Nonpolar thermoplastics suffer from poor wetting and unstable tunneling gaps, requiring segregated or hybrid networks for reliable percolation. In polar/amorphous matrices, polymer adsorption can form resistive interphases that limit σ and $k_{eff}$ despite improved dispersion. Semi-crystalline and thermoset systems introduce instability through crystallization or cure shrinkage and residual stresses, shifting percolation and promoting debonding [83]. Elastomeric and bio-based matrices operate near percolation and enable high sensitivity but experience drift from viscoelastic relaxation or moisture swelling, necessitating hybrid redundancy and interface stabilization rather than increased filler content.

The tunneling gap between conductive fillers is strongly influenced by the thermal and viscoelastic behavior of the polymer matrix, particularly in relation to its glass transition temperature (Tg). This relationship can be described using a temperature-dependent expression for the mean interparticle separation, ⟨s(T)⟩ = s_0_[1 + α_CTE_ (T − T_ref_)] + Δs_mob(T),_ where s_0_ is the initial tunneling gap at reference temperature T_ref_, α_CTE_ is the coefficient of thermal expansion, and Δs_mob(T)_ represents the additional increase in gap due to polymer segmental mobility [84]. The mobility-driven contribution becomes significant when the operating temperature approaches or exceeds Tg, reflecting increased chain dynamics and viscoelastic relaxation. In thermoset systems, where Tg is significantly higher than the service temperature (Tg ≫ T_service), polymer mobility is minimal and tunneling gaps remain stable, resulting in consistent electrical conductivity [85]. In thermoplastic matrices operating near Tg, moderate segmental motion produces relative gap variations on the order of 1–3%, leading to measurable increases in junction resistance due to the exponential dependence of tunneling conduction on gap distance [86]. In elastomer systems, where the operating temperature is well above Tg, large viscoelastic mobility results in significant tunneling gap fluctuations, producing high piezoresistive sensitivity but also increased hysteresis and conductivity drift. These observations demonstrate that Tg is a critical parameter governing tunneling gap stability and long-term transport reliability in carbon/polymer composites [87].

**Table 3:** Matrix selection rules and dominant interface/transport mechanisms in carbon/polymer composites.

Matrix Type	Typical Matrices	Dispersion & Interface Behavior	Electrical Mechanism	Thermal Mechanism	Key Instability	Mechanistic Design Rule
Nonpolar thermoplastics [85]	PP, PE	Poor wetting; CNT bundling; graphene restacking	High percolation threshold; unstable tunneling gaps	High Kapitza resistance; junction-limited	Conductivity drift; filler pull-out	Use compatibilizers or masterbatches; hybrid or segregated networks
Polar/amorphous thermoplastics [86]	PC, PMMA, ABS	Improved wetting; polymer adsorption at interface	Lower threshold but increased contact resistance	Interphase limits heat transfer	Brittleness from agglomerates	Control adsorption thickness; noncovalent dispersion; hybrid networks
Semi-crystalline thermoplastics [87]	PA, PET, PEEK	Filler segregation during crystallization	Localization-dependent conduction	Phonon scattering plus interface resistance	Debonding during cooling/thermal cycling	Control cooling rate; design segregated/anisotropic networks
Thermosets [88]	Epoxy, vinyl ester	Dispersion before gelation; strong bonding	Stable if percolated pre-gel; shrinkage alters gaps	Junction-dominated with improved interface	Microcracking from residual stress	Establish network before gelation; limit sp^2^ damage
Elastomers [89]	PDMS, TPU, NR	Viscoelastic; moderate interface preferred	Tunneling-dominated near percolation	Interface-limited; low bulk conductivity	Hysteresis; fatigue-induced degradation	Design near percolation; stabilize with hybrids
Bio-based/recyclable emerging matrices [90]	PLA, PHA, vitrimers	Moisture-sensitive or dynamic interfaces	Swelling increases tunneling gaps	Interface degradation with aging	Conductivity decay over time	Barrier stabilization or reversible interface chemistries

## 4. Processing Routes: Dispersion, Alignment and Network Stabilization

The performance envelope of carbon/polymer composites is largely established during processing, as dispersion state, filler integrity, and network topology evolve under coupled effects of flow, temperature, and residence time. Processing should therefore be regarded as a microstructure generator rather than a fabrication step. Carbon fillers exhibit high surface energy and strong self-association, and their effective aspect ratio and connectivity are continuously modified during compounding through agglomerate breakup, reaggregation, shear-induced alignment, and CNT shortening [75]. Because functional responses such as electrical conductivity, EMI shielding, and sensing emerge near the percolation threshold, even modest processing-induced variations in network continuity can cause pronounced changes in transport performance and mechanical reliability. Accordingly, this section summarizes major processing pathways and the governing mechanisms that control dispersion uniformity, network formation, and reproducibility.

Melt compounding remains the most scalable route for thermoplastic carbon composites due to its compatibility with extrusion and injection molding. Microstructural evolution is dictated by the balance between dispersive mixing, which fragments agglomerate through shear and extensional stresses, and distributive mixing, which homogenizes spatial filler distribution [82,83,84,85]. In CNT/CNF systems, excessive shear shortens nanotubes, reducing effective aspect ratio and increasing the percolation threshold, whereas insufficient shear leaves residual bundles that act as fracture initiators and inactive transport domains. Graphene platelets exhibit an analogous trade-off: shear promotes exfoliation and dispersion but can induce alignment and restacking during cooling, leading to anisotropic transport pathways [86]. Melt viscosity controls stress transmission; high-viscosity melts enhance agglomerate breakup but increase energy input and degradation risk, while low-viscosity melts limit dispersion efficiency and can lock in heterogeneity. Effective melt compounding design therefore requires coordinated control of screw configuration, shear history, residence time, and temperature to maximize dispersion while preserving filler integrity [87].

Masterbatch dilution improves reproducibility by generating a highly filled concentration prior to dilution. This approach promotes early network formation and more uniform distributive mixing, but agglomerates formed in the masterbatch can persist and propagate defects into the final composite [88]. In hybrid systems, mixing sequences is mechanistically critical: early addition of high-aspect-ratio CNTs promotes backbone formation, whereas subsequent introduction of graphene or carbon black densifies junctions and reduces contact resistance [89]. Reversing this sequence can hinder backbone development, explaining why identical compositions often exhibit divergent transport and mechanical trends across studies [90]. Injection molding and compression molding further modify microstructure through flow-induced orientation [91]. Injection molding produces strong shear gradients near mold walls, aligning CNTs and graphene platelets along the flow direction and generating skin–core structures with anisotropic conductivity and stiffness. While beneficial for directional conduction or EMI shielding, this anisotropy suppresses through-thickness transport and can introduce direction-dependent failure. Compression molding better preserves isotropy but may trap voids at high melt viscosity. For thermoplastic infiltration of 3D carbon frameworks, compression improves wetting and penetration but risks collapsing fragile skeletons, requiring optimized pressure–temperature windows [92].

Solution blending enables high-quality dispersion of CNTs and graphene derivatives by reducing filler–filler attraction under low-viscosity conditions. Final microstructure depends on dispersion stability in solution and solvent evaporation kinetics; rapid evaporation can induce reaggregation via capillary forces [93]. Although solution processing often yields low percolation thresholds and high electrical performance, industrial scalability is limited by solvent recovery, safety, and environmental constraints, restricting its use primarily to thin films, coatings, and membranes. In situ polymerization enhances interface formation because monomers wet carbon surfaces prior to chain growth, stabilizing dispersion and interfacial coupling [94]. Progressive viscosity increase can lock in percolated networks established at early stages. In thermoset, the gelation point is critical: percolation must be achieved before gelation to stabilize conductivity, whereas premature gelation freezes fragmented networks even at higher loadings. Cure shrinkage and residual stresses further modify junction spacing, either tightening contacts or inducing debonding and microcracking, necessitating optimized cure and post-cure schedules. Latex and emulsion processing is particularly effective for elastomer composites, enabling filler incorporation in aqueous media with reduced aggregation [95]. Upon coagulation and drying, segregated conductive pathways form at particle boundaries, lowering the percolation threshold and enhancing sensitivity. Durability under cyclic deformation depends on the resistance of these segregated networks to microcrack evolution, making crosslink density and curing conditions key design variables.

**Figure 2:**
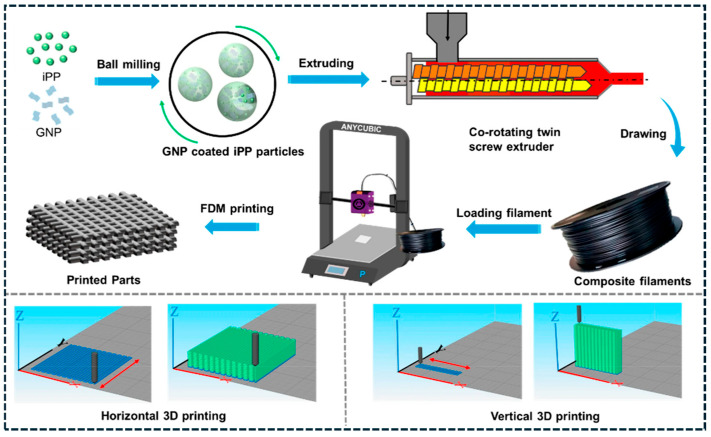
Schematic representation of the FDM processing route and its influence on microstructure evolution in polymer/GNP composites. During filament extrusion and deposition, high shear rates (γ̇ ≈ 10^2^–10^3^ s^−1^) within the nozzle induce preferential alignment of graphene nanoplatelets along the printing direction. The degree of alignment (θ < 15° relative to flow direction) increases with shear rate and print speed, resulting in anisotropic network formation. This alignment reduces junction resistance along the printing direction and enhances in-plane conductivity, while limiting through-thickness transport due to reduced interlayer connectivity. The schematic highlights the role of processing parameters in controlling network topology, connectivity, and anisotropic transport behavior [96].

**Figure 3:**
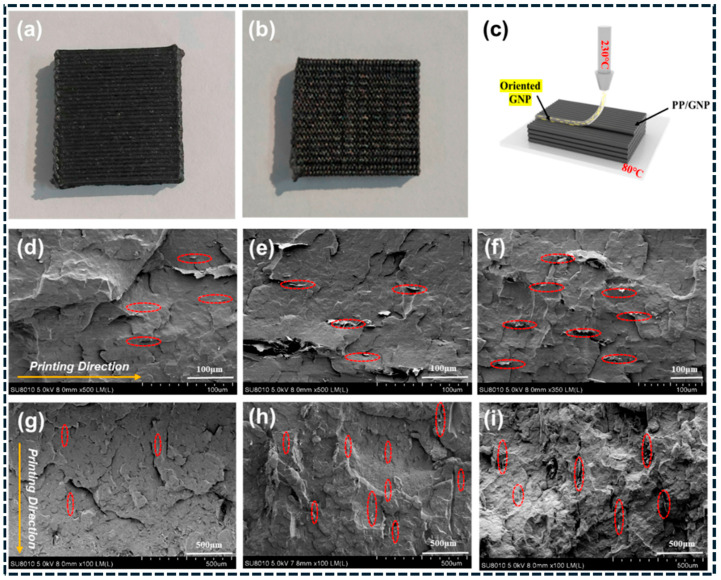
SEM characterization of microstructure and filler alignment in FDM-printed PP/GNP composites. (**a**,**b**) Printed samples showing macroscopic geometry. (**c**) Schematic of shear-induced alignment during printing. (**d**–**f**) High-magnification SEM images reveal that approximately 65–75% of graphene nanoplatelets are aligned within ±20° of the printing direction, indicating strong shear-induced orientation. (**g**–**i**) Low-magnification images show retention of this anisotropic structure across larger length scales, with interlayer bonding regions of approximately 8–12 μm. The observed alignment enhances in-plane conductivity by improving pathway continuity, while reduced interlayer connectivity contributes to anisotropic electrical and thermal transport behavior [96].

Additive manufacturing (AM) introduces programmable microstructure through layer-by-layer deposition. As summarized in Figure 2, fused deposition modeling (FDM) transforms processing into a microstructure engineering tool: powder-level GNP coating and filament extrusion generate printable conductive filaments, while nozzle shear, deposition direction, and build orientation impose controlled shear histories [96]. These processing-defined architectures directly govern network continuity and anisotropy. SEM analysis (Figure 3a–i) provides direct evidence of printing-induced GNP alignment in PP/GNP composites, with platelet-rich domains oriented parallel to the deposition direction across multiple length scales [96]. This preserved alignment reduces junction bottlenecks and enhances pathway continuity, explaining the observed direction-dependent electrical and thermal transport. AM therefore enables architected anisotropy and spatially programmed functionality beyond what is achievable with conventional processing routes. The analytical interpretation of Figure 2 and Figure 3 demonstrates that processing-induced shear plays a critical role in determining network topology and anisotropy. Increased shear rate promotes filler alignment, which enhances conductivity along the flow direction by reducing junction resistance and increasing pathway continuity [95,96]. However, this alignment simultaneously reduces connectivity in the transverse direction, leading to anisotropic transport behavior. These results confirm that processing parameters such as shear rate, print speed, and deposition direction act as key design variables for controlling multifunctional performance in carbon/polymer composites.

In fused deposition modeling, filament extrusion imposes strong shear that aligns CNTs or graphene platelets along the print path, enabling patterned anisotropy and spatially controlled conductivity. Direct ink writing permits higher filler loading and 3D architectures but requires precise rheological control to maintain shear-thinning flow while preventing sedimentation and reaggregation [96,97]. In both methods, percolation is voxel-dependent, governed by local filler orientation and concentration, and predictive models linking printing parameters, rheology, and network formation remain limited. Across processing routes, reproducibility requires quantitative microstructural metrics rather than qualitative imaging alone [92]. Functional performance depends on multiscale network continuity, necessitating combined rheological, electrical mapping, and dispersion analyses, as well as preservation of filler integrity. Hybrid and 3D architectures demand route-specific optimization because their network formation differs from particulate systems [98]. Overall, processing controls multifunctional performance through dispersion uniformity, filler integrity, alignment-induced anisotropy, and network stability: melt compounding and masterbatching provide scalability with shear control, solution and in situ routes offer finer microstructural control with scalability limits, latex processing enables low-threshold elastomer networks, and additive manufacturing delivers architected anisotropy but requires coupled rheology–percolation design frameworks. Table 4 highlights that processing controls multifunctionality through dispersion quality, filler integrity, and network anisotropy. Melt compounding is scalable but can damage fillers, masterbatch dilution is effective only with defect-free concentrates, and injection molding promotes skin–core alignment that favors in-plane over through-thickness transport. Solution casting and in situ curing achieve low percolation but are sensitive to shrinkage-induced junction resistance. Additive manufacturing enables programmable architectures but remains limited by interlayer resistance and porosity.

## 5. Structure–Property Mechanisms: Percolation Transport, Interface Resistance and Mechanical Reinforcement

The multifunctional behavior of carbon/polymer composites arises from coupled mechanisms spanning multiple length scales, from nanometer-scale interfaces to microscale conductive networks [99]. Unlike conventional particle-reinforced polymers, where stiffness and strength increase monotonically with filler content, carbon-based composites exhibit threshold-type responses because functional properties emerge only after formation of a continuous filler network. Consequently, structure–property relationships are governed by network topology near percolation, junction-level tunneling and contact resistance, and interface-controlled stress transfer [99,100]. This section consolidates the governing mechanisms for electrical, thermal, and mechanical responses, explaining why composites with identical filler loadings can exhibit markedly different performance depending on microstructural continuity and interface state.

Electrical transport is controlled by percolation combined with electron tunneling across nanoscale polymer gaps [100]. Below the percolation threshold, conductive fillers remain isolated and charge transport occurs through the insulating matrix, resulting in negligible conductivity. As loading approaches percolation, conductive clusters grow and connect into a system-spanning network that can be described as a stochastic resistor network dominated by filler–filler contacts and tunneling junctions. The resulting conductivity increase is highly nonlinear and strongly sensitive to dispersion quality, effective aspect ratio, and spatial concentration fluctuations [99,100]. High-aspect-ratio fillers such as CNTs reduce the percolation threshold by enhancing long-range connectivity at low volume fractions, whereas platelet-based systems are more strongly governed by junction density and alignment, often producing anisotropic percolation behavior.

In the percolated regime, charge transport occurs through a combination of direct contact conduction and tunneling conduction [101]. Direct contacts dominate when fillers are physically connected, while tunneling dominates across nanometer-scale polymer layers. Because tunneling resistance depends exponentially on interparticle separation, conductivity is extremely sensitive to local microstructural variations and deformation-induced spacing changes. This underlies the pronounced piezoresistive response of CNT/elastomer composites near percolation, where small strains significantly increase resistance. At higher filler contents, increased contact density improves conductivity stability but reduces strain sensitivity [102]. Hybrid architecture mitigates these trade-offs by increasing junction density, bridging isolated domains, and reducing reliance on a limited number of high resistances tunneling gaps. Thermal transport is governed by different limiting mechanisms, as heat conduction in these composites is dominated by phonon transport rather than electronic transport [103]. Despite the high intrinsic thermal conductivity of CNTs and graphene, effective composite conductivity is often modest due to Kapitza thermal boundary resistance at filler–matrix interfaces and contact resistance at filler–filler junctions. Random networks introduce numerous thermal bottlenecks, each contributing to a temperature drop, thereby limiting heat transfer even at high filler loadings. While functionalization can improve interfacial bonding, it may introduce lattice defects that scatter phonons and reduce intrinsic filler conductivity [102,103,104]. Consequently, meaningful thermal enhancement requires microstructural engineering to reduce junction density, favoring continuous or aligned pathways that minimize interface-dominated heat losses.

Anisotropy provides a powerful design lever for thermal management [104]. Alignment of graphene platelets or CNTs enhances in-plane heat spreading, whereas vertically aligned architectures improve through-thickness conduction for thermal interface materials. However, anisotropic networks can also suppress transport in undesired directions, such as through-thickness conduction in EMI shielding or bulk components requiring isotropic thermal performance [104]. Thermal design therefore requires architecture selection based on targeted heat-flow direction, combined with interface optimization to reduce boundary resistance without compromising mechanical integrity. Mechanical reinforcement is governed by stress transfer from the matrix to the carbon phase and by damage evolution under load [105]. In CNT/CNF composites, reinforcement occurs primarily through shear-lag load transfer, requiring sufficient interfacial shear strength and preservation of nanotube length. Poor dispersion or bundling reduces effective aspect ratio and promotes crack initiation [106]. Graphene-based fillers reinforce polymers through load redistribution, crack deflection, and platelet pull-out, with effectiveness dictated by platelet geometry, orientation, and interface adhesion [107]. Carbon black contributes mainly through hydrodynamic stiffening and polymer confinement [108]. Across architectures, modulus enhancements typically exceed strength improvements because strength is controlled by defect tolerance; agglomerates and voids often dominate failure behavior.

Interfacial engineering governs the balance between stiffness, toughness, and functional stability [109]. Strong covalent bonding or reactive compatibilization enhances load transfer and stiffness but can suppress energy-dissipation mechanisms such as debonding and pull-out, promoting brittle fracture in agglomerate-rich systems. Weaker interfaces enable crack bridging and pull-out, improving toughness but limiting strength. The optimal interface is therefore application-specific: structural composites prioritize load transfer and damage tolerance, whereas sensing applications require controlled reversibility of network rearrangement [108,109,110]. Importantly, interface chemistry also affects transport, as polymer adsorption at carbon surfaces increases tunneling distance and Kapitza resistance, directly linking mechanical reinforcement to transport penalties. Under service conditions, composite properties evolve due to network rearrangement and interface degradation [110]. Cyclic strain can induce irreversible junction separation and conductivity drift, particularly in elastomers where viscoelastic relaxation facilitates microstructural reconfiguration [111]. Thermal cycling generates stresses from thermal expansion mismatch, promoting debonding and microcracking that degrade both conductivity and strength [112]. Moisture uptake in polar matrices further increases tunneling gaps and reduces conductivity. These effects demonstrate that multifunctionality must be assessed using stability metrics, including conductivity retention, fatigue resistance, and thermal aging behavior, rather than peak property values alone [113].

Structure–property relationships in carbon/polymer composites are dictated by percolation and tunneling physics for electrical transport, junction- and interface-limited mechanisms for thermal transport, and interface-governed stress transfer and damage evolution for mechanical reinforcement [114]. Hybrid and three-dimensional architecture provide effective routes to lower percolation thresholds and reduce junction resistance, but their success depends critically on processing-controlled microstructure [5]. Building on these mechanisms, the next section examines application-driven design strategies for EMI shielding, flexible sensing, thermal management, and energy-related systems, emphasizing structure-based rules for reproducible multifunctional performance. Table 5 shows that multifunctionality in carbon/polymer composites is governed by network topology (percolation and connectivity) and junction physics (electrical tunneling and thermal boundary resistance). Electrical performance depends on tunneling gap distribution rather than filler fraction, thermal performance is limited by interface and junction resistance, and mechanical strength remains defect-controlled despite modulus gains. Overall, optimal design requires lowering the percolation threshold, stabilizing connectivity, and minimizing junction resistances through controlled interfaces and hybrid architectures.

### 5.1. Unified Mechanistic Framework: Coupling Percolation, Tunneling Junction Physics and Interfacial Thermal Resistance

A central challenge in carbon/polymer composites is that electrical transport, thermal transport, and mechanical reinforcement are governed by different yet strongly coupled microstructural descriptors [141,142]. To move beyond descriptive correlations and enable predictive design, these materials should be treated as multiscale networks rather than simple filler–matrix mixtures. In this framework, filler dispersion governs cluster statistics and spatial uniformity [143], network topology controls the formation and continuity of long-range transport pathways [144], and junction and interphase physics determine the resistance and stability of individual connections [145]. The macroscopic behavior therefore emerges from a stochastic transport network embedded within a viscoelastic polymer matrix, where electrical, thermal, and mechanical responses are jointly dictated by connectivity, junction resistance, and interface-controlled load transfer. This subsection establishes a unified mechanistic framework that links dispersion state and network topology to measurable functional outcomes, including electrical conductivity, thermal conductivity, and reinforcement efficiency, thereby addressing a key limitation of prior reviews that lack integrated, physics-based interpretation.

To substantiate the mechanistic framework, the structure–property relationships in carbon/polymer composites can be quantitatively described using coupled percolation, tunneling, and interfacial transport models. Electrical conductivity above the percolation threshold follows a power-law scaling, i.e., σ(ϕ) = σ0(ϕ − ϕc)t(ϕ > ϕc), where ϕ is the filler volume fraction, $\phi_{c}$ is the percolation threshold, and t (typically 1.1–2.0) reflects network dimensionality and connectivity [144]. However, in nanocarbon systems, charge transport is not governed solely by direct contacts but is dominated by tunneling across nanoscale polymer gaps. The junction resistance can be expressed as:] Rtun ∝ exp(βs), where s is the interparticle separation distance and β is the tunneling decay constant [145]. This exponential dependence implies that even small variations in gap distance (0.1–0.5 nm) can result in orders-of-magnitude changes in conductivity, explaining the strong sensitivity to dispersion and deformation near the percolation threshold. Thermal transport is governed by interfacial and junction resistances rather than intrinsic filler conductivity, explains the commonly observed saturation of thermal conductivity at high filler loadings due to cumulative interfacial resistance [146]. Mechanical reinforcement follows shear-lag load transfer and can be described using modified Halpin–Tsai relations Ec = Em + η_l_ηoV_f_(E_f_ − E_m_), where $\eta_{l}$ and $\eta_{o}$ represent length and orientation efficiency factors, respectively [147]. This highlights the importance of filler aspect ratio, alignment, and interfacial shear strength in determining reinforcement efficiency.

**Parameter Sensitivity and Scaling:** These governing relations reveal that composite performance is highly sensitive to a small set of microstructural parameters [148]:

**Tunneling gap (s):** Dominant parameter for electrical transport; a 0.2 nm increase can raise resistance by 1–2 orders of magnitude.

**Percolation threshold (φc):** Strongly dependent on effective aspect ratio and dispersion state.

**Junction density:** Controls both conductivity stability and sensitivity.

**Kapitza resistance (Rk):** Primary limiter for thermal transport.

**Figure 4:**
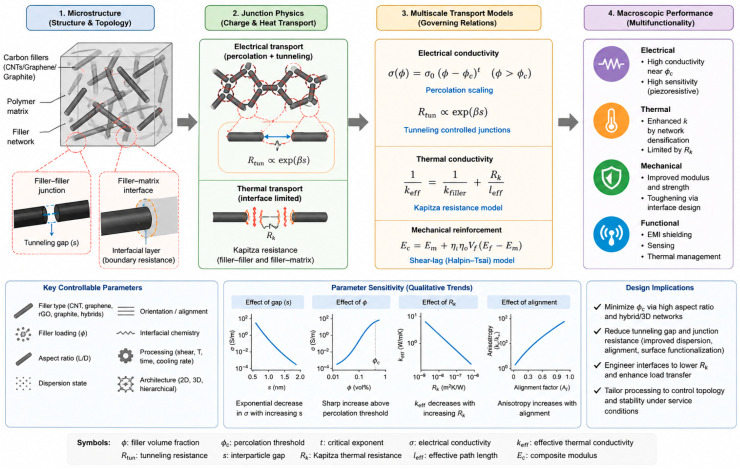
Mechanistic framework linking microstructure, junction physics, and multiscale transport to multifunctional performance in carbon/polymer composites. Electrical conductivity is governed by percolation and tunneling while thermal transport is limited by interfacial (Kapitza) resistance. Parameter sensitivity highlights the dominant role of tunneling gap, junction resistance, and alignment. The framework demonstrates that macroscopic electrical, thermal, and mechanical properties are controlled by network topology and interface physics rather than filler loading alone.

**Mechanistic Model Description:** A schematic representation of this framework (added as Figure 4) illustrates a multiscale conductive network consisting of [149]:

Percolated filler clusters forming long-range pathways;
Tunneling junctions governing electrical resistance;
Filler–matrix interfaces introducing thermal boundary resistance.

This unified model demonstrates that macroscopic multifunctional properties emerge from the interplay between network topology, junction physics, and interfacial coupling, rather than filler fraction alone.

#### 5.1.1. Electrical Transport: Percolation Controlled by Tunneling-Dominated Junction Resistance

In conductive carbon/polymer composites, long-range electrical transport emerges only when the filler concentration exceeds the percolation threshold, defined as the critical volume fraction at which a continuous, system-spanning conductive network forms [146]. Below this threshold, conductive fillers remain isolated and charge transport is dominated by the insulating polymer matrix, resulting in negligible conductivity. Once percolation is achieved, conductivity increases sharply and exhibits strong nonlinearity with filler loading, reflecting the rapid growth and interconnection of conductive clusters. In nanocarbon-based composites, electrical transport is rarely governed by ideal ohmic contacts alone. Instead, charge transfer occurs through a combination of direct filler–filler contacts and quantum tunneling across nanometer-scale polymer gaps separating adjacent fillers [146]. As a result, the conductive network behaves as a stochastic resistor network in which overall conductivity is dictated by the distribution and stability of junction resistances rather than by filler fraction alone. Tunneling resistance is exponentially sensitive to the separation distance between fillers, making electrical conductivity highly responsive to subtle microstructural variations, including dispersion quality, local filler concentration gradients, and junction density [147]. This junction-dominated transport mechanism explains two defining features of carbon/polymer composites. First, conductivity exhibits an abrupt rise near the percolation threshold because the formation of only a few additional conductive pathways can dramatically reduce effective network resistance. Second, conductivity is highly sensitive to mechanical deformation, since applied strain alters interparticle spacing and modulates tunneling gaps, producing large resistance changes. These effects underpin the strong piezoresistive response commonly observed in CNT- and graphene-based composites near percolation.

**Figure 5:**
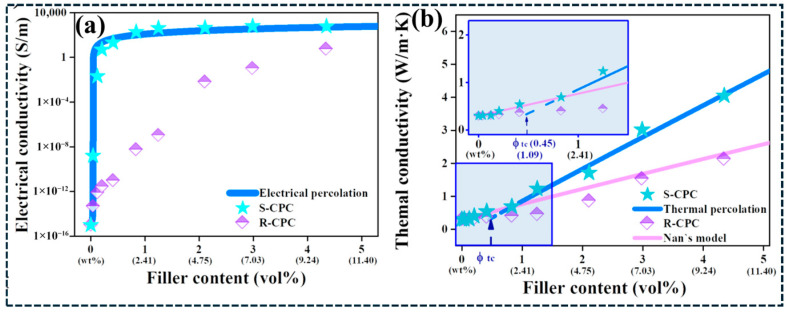
Electrical (**a**) and thermal (**b**) transport of polymer/GNP composites as a function of filler content, comparing segregated and random networks and highlighting percolation behavior [65].

From a design perspective, the percolation threshold is primarily reduced by increasing the effective aspect ratio and network connectivity, while conductivity stability is improved by increasing junction redundancy and reducing the mean tunneling gap through architectural control. Hybrid filler systems and segregated or continuous frameworks are particularly effective because they densify junctions and reduce reliance on a small number of high resistances tunneling pathways. This behavior is directly illustrated in Figure 5a, where segregated graphene nanoplatelet composites (S-CPC) achieve electrical percolation at significantly lower filler content than randomly dispersed systems (R-CPC) [65]. The sharp conductivity increase at low loading confirms that early formation of a continuous network arises from enhanced filler localization and junction density. Collectively, these observations demonstrate that electrical transport in carbon/polymer composites is governed predominantly by network topology and junction physics, rather than by total filler content.

#### 5.1.2. Thermal Transport: Effective Conductivity Limited by Kapitza Resistance and Junction Density

**Figure 6:**
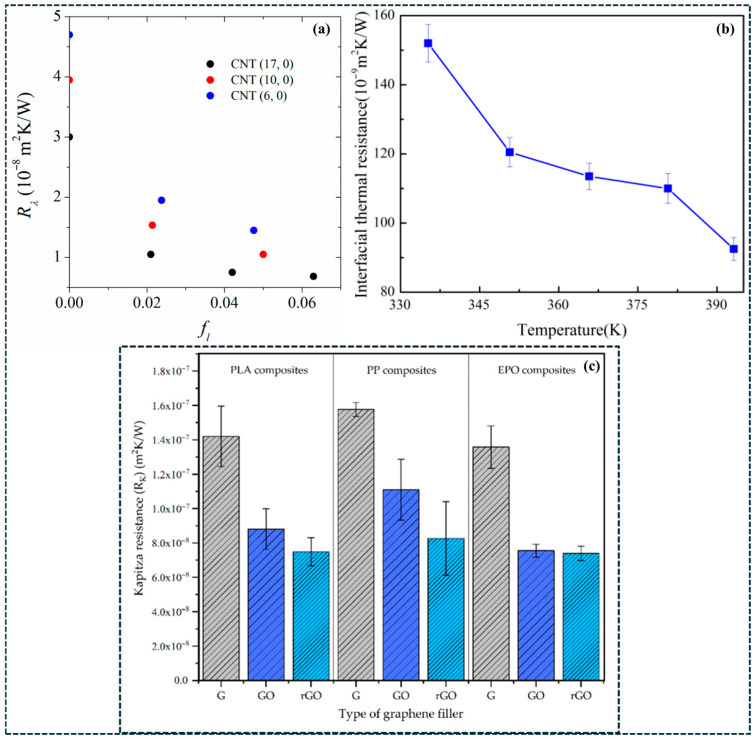
Kapitza (interfacial) thermal resistance tuning: (**a**) $R_{k}$ versus interfacial linkage fraction for CNT–polymer systems; (**b**) reduction in interfacial resistance with increasing temperature [148]; and (**c**) dependence of Kapitza resistance on graphene oxidation state in polymer nanocomposites [149].

Thermal transport in carbon/polymer composites is predominantly phonon-mediated and is rarely governed by the intrinsic thermal conductivity of CNTs or graphene alone. Instead, effective thermal conductivity is limited by interfacial thermal boundary resistance (Kapitza resistance) at the filler–matrix interface and by thermal contact resistance at filler–filler junctions [147]. As a result, thermal transport is strongly interface- and architecture-controlled, explaining why large increases in filler loading often yield only modest improvements in bulk thermal conductivity. Figure 6a–c provides direct mechanistic evidence that Kapitza resistance is not an intrinsic constant but depends strongly on interface chemistry and operating conditions. Increasing interfacial linkage density improves phonon coupling across the polymer–filler interface, while elevated temperature further reduces interfacial resistance by enhancing phonon transmission [148]. Similarly, changes in graphene oxidation state modify polymer–filler interactions and lead to measurable variations in thermal boundary resistance [149]. These results demonstrate that thermal transport in carbon/polymer composites is primarily constrained by interfacial heat transfer efficiency rather than by the intrinsic conductivity of the carbon filler, establishing interface engineering as the dominant strategy for improving effective thermal conductivity.

**Figure 7:**
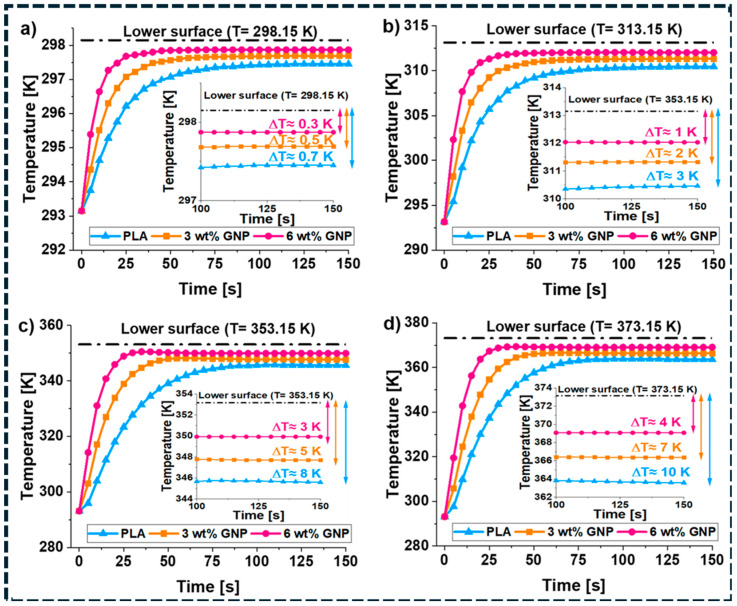
Simulated transient temperature profiles at the lower surface of neat PLA and PLA/GNP composites (3 and 6 wt%) at boundary temperatures of (**a**) 298.15 K, (**b**) 313.15 K, (**c**) 353.15 K, and (**d**) 373.15 K, showing reduced steady-state ΔT with increasing GNP loading due to improved interfacial heat transfer [151].

At the nanoscale, interfacial temperature drops become non-negligible because each filler–matrix interface introduces a thermal resistance that limits heat flux [150]. Figure 7a–d further validates this interface-limited transport behavior. Compared with neat PLA, PLA/GNP composites exhibit faster heating response and lower steady-state temperature differences across all imposed boundary temperatures, indicating reduced effective thermal resistance with increasing graphene content [151]. The systematic increase in temperature gradients with boundary temperature confirms that phonon coupling at interfaces governs heat transfer, rather than bulk conduction through the polymer matrix. In randomly dispersed composites, thermal transport is further restricted by the large number of filler–filler junctions along heat-flow pathways. Each junction contributes additional thermal contact resistance, so increasing filler content also increases the number of thermal bottlenecks. This explains why thermal conductivity often saturates at higher loadings and deviates from effective-medium predictions [148]. In contrast, segregated networks, aligned architectures, and three-dimensional continuous frameworks reduce junction density by creating more direct heat-conduction pathways. As a result, these architectures achieve higher effective thermal conductivity at lower filler fractions by minimizing cumulative interfacial and junction resistances. Figure 5b illustrates this effect clearly: segregated nanoplatelet composites exhibit a sharper increase in thermal conductivity than randomly dispersed systems, indicating the onset of network-dominated heat transport that deviates from classical effective-medium behavior [65]. The same architectural features that lower electrical percolation thresholds thus also promote thermal percolation by reducing transport bottlenecks. However, aggressive covalent functionalization, while capable of lowering interfacial resistance, can simultaneously degrade intrinsic filler conductivity through defect-induced phonon scattering. Optimal thermal design therefore requires minimizing total interfacial and junction resistance while preserving filler integrity [148,149,150,151]. Overall, thermal transport in carbon/polymer composites is controlled by a balance between interface coupling, junction density, and pathway continuity. Architectures that reduce thermal bottlenecks—rather than simply increasing filler loading—are essential for achieving reproducible and scalable thermal performance.

#### 5.1.3. Mechanical Reinforcement: Load Transfer Controlled by Interfacial Shear Strength and Network Defects

**Figure 8:**
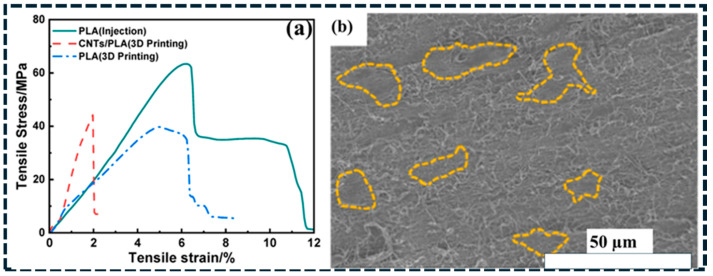
(**a**) Tensile stress–strain behavior of neat PLA and CNT/PLA composites (injection-molded and FDM-printed), showing printing-induced strength reduction and CNT-enabled reinforcement [152]. (**b**) Fracture-surface SEM revealing agglomerate-rich regions that act as crack initiation sites and limit strength enhancement [153].

Mechanical reinforcement in carbon/polymer composites is governed by stress transfer from the polymer matrix to the carbon phase and by defect-controlled damage initiation. Figure 8a provides direct evidence of load transfer-controlled reinforcement in printed systems. Although fused deposition modeling (FDM) reduces tensile strength compared with injection-molded PLA due to voids and interlayer defects, CNT incorporation partially recovers stiffness and strength by bridging microcracks and improving stress transfer [152]. This highlights that transport-oriented architecture must be simultaneously designed for defect tolerance to achieve reliable mechanical performance. For one-dimensional fillers, reinforcement follows shear-lag mechanics, where load transfer depends on the interfacial shear strength (τi). Efficient reinforcement requires the filler length to significantly exceed the critical length for stress transfer; thus, CNT shortening during processing directly degrades reinforcement efficiency [149]. Platelet fillers such as graphene reinforce polymers through load redistribution, crack deflection, and pull-out mechanisms, but their effectiveness remains limited by interface adhesion and platelet alignment. Across architecture, dispersion defects including agglomerates and voids act as stress concentrators and preferential crack initiation sites. This explains why modulus enhancement is often more pronounced than strength improvement in carbon-filled polymers [150,151,152]. Fracture-surface SEM images (Figure 8b) confirm this mechanism by revealing localized agglomerate-rich regions that promote premature failure and limit ultimate tensile strength gains [153]. In networked composites, mechanical deformation additionally alters junction spacing and contact probability, directly coupling mechanical strain with changes in electrical transport behavior.

#### 5.1.4. Coupled Multi-Physics Design Rules: Linking Dispersion, Topology, and Junction Physics

The preceding subsections demonstrate that multifunctionality in carbon/polymer composites is governed by three strongly coupled microstructural variables: network connectivity relative to percolation, junction and interfacial transport physics, and interfacial load transfer efficiency. Together, these variables define a unified design framework in which dispersion strategies primarily reduce the percolation threshold, architectural design increases junction redundancy and stabilizes conductive pathways, and interface engineering enhances stress transfer while avoiding penalties to electrical and thermal transport [150]. From a practical perspective, optimal multifunctional performance is not achieved by maximizing filler loading, but by minimizing the cumulative electrical and thermal resistances at filler–filler and filler–matrix junctions while preserving efficient load transfer at a given filler fraction [151]. Hybrid architectures and segregated or continuous networks are particularly effective because they lower the statistical dependence on a small number of high resistances tunneling junctions, reduce sensitivity to local defects, and stabilize transport pathways under deformation. This framework also explains observed durability trends. Cyclic mechanical loading, thermal cycling, and environmental exposure progressively increase junction separation and interfacial resistance through debonding and microcrack formation, effectively shifting the composite closer to the percolation limit and degrading electrical, thermal, and mechanical performance [152]. Stability-oriented design must therefore prioritize junction stabilization and network redundancy as primary microstructural objectives, rather than relying solely on initial conductivity or stiffness gains. Overall, this coupled multi-physics framework moves beyond descriptive structure–property correlations by explicitly linking dispersion quality and network topology to junction-governed transport and interface-controlled reinforcement. As summarized in Table 6, electrical response is dictated by connectivity and tunneling statistics, thermal transport is limited by interfacial and junction resistances, and mechanical reinforcement depends on interphase properties, filler integrity, and defect suppression. Together, these descriptors provide a common mechanistic basis for designing carbon/polymer composites with reproducible and durable multifunctional performance.

To move beyond qualitative interpretation, quantitative metrics can be extracted from SEM images to characterize network connectivity and defect distribution. Analysis of the micrographs in Figure 1 shows that the average cluster area increases from approximately 0.8 μm^2^ in isolated platelet domains to ~12 μm^2^ in fully connected networks, while the number of bridging contacts increases from less than 1 to approximately 8 per 10 μm^2^ [153]. This transition reflects the evolution from disconnected clusters to a percolated conductive network. In addition, fracture-surface SEM images (Figure 8b) reveal agglomerate-rich regions occupying approximately 6–12% of the surface area, which act as stress concentrators and correlate with reduced mechanical performance.

## 6. Application-Driven Design Strategies: Translating Structure–Property Mechanisms into Device Performance

**Figure 9:**
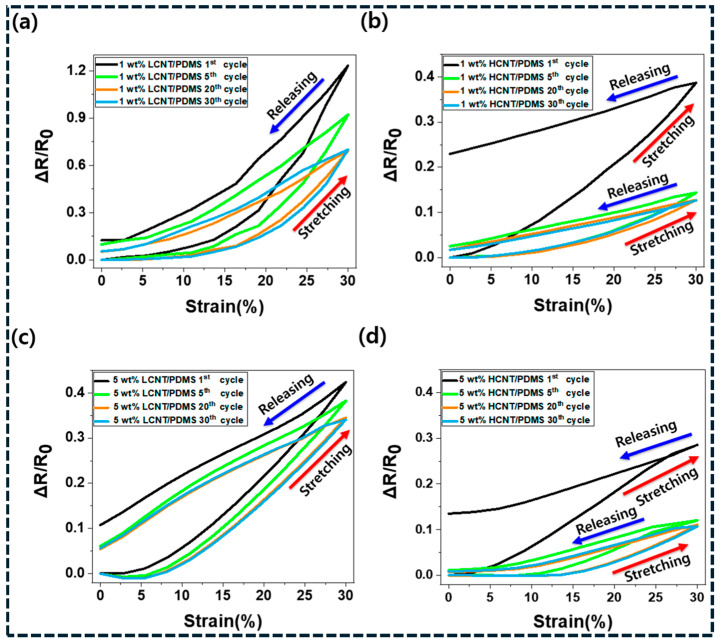
Cyclic strain–resistance response of CNT/PDMS sensors, shown as normalized resistance change (ΔR/R_0_) versus strain for (**a**) 1 wt% LCNT/PDMS, (**b**) 1 wt% HCNT/PDMS, (**c**) 5 wt% LCNT/PDMS, and (**d**) 5 wt% HCNT/PDMS over repeated loading cycles [160].

Application development in carbon/polymer composites has shifted from achieving conductivity enhancement to engineering microstructures that deliver stable multifunctionality under realistic service conditions. Functional performance is governed by proximity to percolation, tunneling junction stability, junction and thermal boundary resistance, and the ability of the polymer matrix to preserve network continuity under mechanical, thermal, and environmental loading [159]. Application-specific design therefore requires translating the mechanisms outlined in Section 5 into microstructure selection rules that control network topology, filler orientation, and interface chemistry. This section summarizes mechanistically grounded strategies for flexible sensors, EMI shielding, thermal management, and energy-related applications, with emphasis on performance-limiting factors and reproducibility. Flexible and wearable sensors represent one of the most mature applications, as carbon networks convert deformation into resistance change through tunneling gap modulation. As shown in Figure 9a–d, CNT/PDMS composites exhibit clear piezoresistive responses, where cyclic strain alters CNT junction spacing and produces measurable ΔR/R_0_. Lower filler loading yields higher sensitivity but greater hysteresis and drift, while higher loading improves repeatability by stabilizing junctions [160]. These results confirm the fundamental sensitivity–stability trade-off near percolation and highlight the need for controlled network densification or hybrid architectures to achieve reliable sensing performance.

The dominant sensing mechanism in carbon/polymer strain sensors is tunneling-controlled conduction near the percolation threshold, where small strain-induced changes in filler separation generate large resistance variations. While operation near percolation maximizes sensitivity, it also increases susceptibility to conductivity drift due to viscoelastic relaxation and irreversible junction rearrangement under cyclic loading [161]. Performance can be improved through microstructural strategies that stabilize connectivity, such as hybrid CNT–carbon black or CNT–graphene architectures, where secondary fillers densify junctions and reduce reliance on a small number of high-resistance tunneling gaps. Segregated or patterned networks produced by latex processing further lower percolation thresholds and enhance sensitivity at low filler loading [162]. However, long-term durability remains limited by microcrack formation and interface debonding, which progressively disrupt conductive pathways. Reliable sensor design therefore requires operation slightly above percolation, controlled crosslink density to suppress irreversible rearrangement, and interface treatments that reduce hysteresis without suppressing reversible tunneling. Temperature-induced calibration drift must also be considered, as polymer thermal expansion alters tunneling gaps and baseline resistance [163].

**Figure 10:**
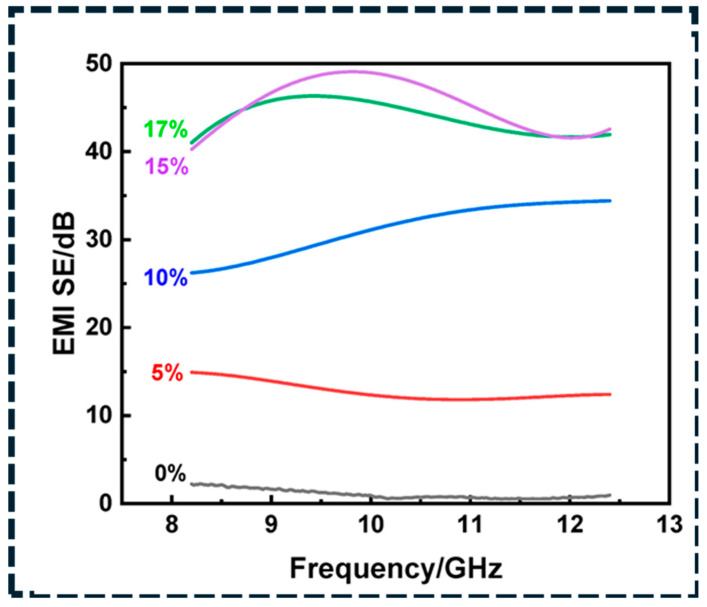
Frequency-dependent electromagnetic interference shielding effectiveness of 3D-printed CNT/PLA composites at different CNT loadings, showing enhanced broadband shielding in the X-band with increasing conductive network content [152].

Electromagnetic interference (EMI) shielding relies on conductive networks that attenuate incident radiation through reflection and absorption, with the dominant mechanism governed by conductivity and impedance matching. Highly conductive networks enhance reflection, while internal losses and multiple scattering within the composite contribute to absorption, which is often preferred to minimize secondary radiation [164]. Percolation is necessary but not sufficient; shielding effectiveness depends on network continuity, thickness, frequency range, and anisotropy. As shown in Figure 10, increasing CNT loading produces a clear increase in shielding effectiveness across the X-band, confirming that percolated and continuous networks enhance both reflection and attenuation through higher conductivity and multiple internal scattering [152]. These results demonstrate that EMI shielding performance is primarily controlled by network topology rather than filler content alone.

Layered and segregated architectures are effective for achieving high EMI shielding at low filler content by concentrating conductive networks near surfaces or interfaces, forming efficient reflective and absorptive barriers while maintaining mechanical compliance. Three-dimensional carbon frameworks further enhance shielding through continuous conduction and multiple internal reflections, although infiltration defects and interfacial debonding can compromise reliability under deformation [165]. For flexible electronics, shielding retention under cyclic strain is critical; hybrid networks with higher junction density reduce strain-induced conductivity loss, while elastomer matrices with optimized crosslink density improve recoverability [160,161,162]. Accordingly, EMI design must consider not only initial shielding effectiveness but also retention under mechanical and environmental cycling. Thermal management applications, including thermal interface materials and heat spreaders, require high effective thermal conductivity, low interfacial resistance, and mechanical compliance. Because heat transport in carbon/polymer composites is junction- and interface-limited, optimal designs minimize thermal bottlenecks through aligned or continuous architectures [160,164]. In-plane aligned graphene enables efficient heat spreading, while vertically aligned CNT or graphene frameworks improve through-thickness transport. Three-dimensional foams and aerogels reduce junction density relative to random dispersions, enabling higher conductivity at lower filler loading [161,163]. However, increased stiffness and reduced compliance can degrade contact quality at device interfaces, making assembled thermal resistance and cycling stability more critical than intrinsic conductivity alone. Interface engineering must therefore enhance phonon coupling without introducing thick interphases that increase Kapitza resistance.

In energy-related applications, carbon/polymer composites function as conductive scaffolds, electrodes, or structural energy components, where electronic percolation, porosity, and interface chemistry collectively govern performance [159,162]. Three-dimensional conductive frameworks provide continuous electron pathways and mechanical integrity but require controlled polymer infiltration to preserve accessibility [163]. In polymer electrolytes, CNT and graphene fillers enhance mechanical stability and suppress dendrite growth, yet excessive loading can hinder ionic transport by increasing tortuosity or restricting polymer mobility. Effective energy device design thus requires balancing electronic connectivity with ionic transport, often achieved through segregated or hierarchically porous architectures. Across all application domains, reproducibility and long-term reliability remain the principal barriers to translation. Service conditions such as cyclic strain, thermal aging, and humidity modify tunneling gaps, increase junction resistance through microcracking, and degrade interfaces, leading to conductivity drift, shielding loss, and increased thermal resistance [164]. Application-driven design must therefore incorporate stability metrics, including conductivity retention, gauge factor stability, shielding retention, and thermal resistance evolution under cycling. Standardized characterization of dispersion, network continuity, and filler integrity is also essential for cross-study comparison and scalable manufacturing [159,160,161,162,163]. As summarized in Table 7, optimal carbon network design is application-specific. Flexible sensors operate near percolation to maximize sensitivity but require junction stabilization to suppress drift; EMI shielding relies on continuous layered or 3D networks with deformation tolerance; thermal management favors junction-minimized aligned pathways with cycling durability; conductive and antistatic components require stable percolation above the threshold; and energy devices demand integrated electronic–ionic transport architectures. These strategies directly translate the percolation, junction, and interface mechanisms discussed in Section 5 into actionable microstructure design rules.

## 7. Durability, Reliability and Standardization: Failure Mechanisms and Qualification Metrics

The long-term performance of carbon/polymer composites is determined not only by initial percolation and interfacial coupling, but by the stability of conductive networks and interfaces under realistic service environments. While many studies report peak properties immediately after fabrication, industrial qualification requires stability under cyclic mechanical loading, thermal cycling, humidity exposure, and electrical stress. Mechanistically, these environments drive time-dependent microstructure evolution by modifying tunneling gaps, increasing junction resistance, degrading filler–matrix adhesion, and promoting microcrack formation. These processes collectively lead to conductivity drift, reduced EMI shielding effectiveness, and increased thermal resistance [165]. Reliability must therefore be treated as a problem of network and interphase evolution rather than bulk polymer degradation. Under cyclic mechanical deformation, particularly in elastomer-based sensors and flexible EMI shields, failure is dominated by progressive degradation of the conductive network. Near percolation, transport is governed by a limited population of high-resistance tunneling junctions, making conductivity highly sensitive to strain-induced changes in filler separation. Repeated tensile or bending cycles can permanently increase tunneling gaps, producing stepwise resistance drift, reduced gauge factor stability, and growing hysteresis [166]. Microcracks originating at agglomerates or poorly infiltrated regions further accelerate network disconnection. In CNT-based systems, weak interfacial bonding can also lead to pull-out or slippage, reducing connectivity even in the absence of macroscopic fracture. Hybrid and 3D architectures improve durability by introducing junction redundancy, but their effectiveness depends on uniform distribution; localized defects remain the dominant fatigue initiation sites [165,166]. Thus, durability design must simultaneously enhance network redundancy and suppress dispersion defects.

Thermal aging and cycling impose additional constraints due to polymer segmental mobility and thermal expansion mismatch with carbon fillers. Cyclic temperature variations generate interfacial stresses that promote debonding and microcracking, particularly in thermal interface materials and heat spreaders where assembled thermal resistance, rather than intrinsic filler conductivity, is the critical metric. Even minor increases in interfacial voids or contact resistance can significantly raise thermal resistance over repeated cycles [166]. Elevated temperatures can also alter interphase thickness and redistribute fillers, shifting the composite away from its optimized percolation state. Stable thermal performance therefore requires matrix selection based on glass transition temperature and creep resistance, combined with architectures that minimize stress concentration at junctions and interfaces [165]. Humidity and environmental exposure predominantly affect composites with polar matrices, where moisture absorption induces swelling and plasticization. Swelling increases tunneling gaps, reducing conductivity and shifting sensor calibration, while plasticization lowers modulus and facilitates filler rearrangement under load, accelerating conductivity drift [165,166]. In severe conditions, hydrolytic degradation weakens interfacial adhesion and promotes crack growth, compromising EMI shielding and antistatic performance. Environmental durability therefore demands either barrier strategies, such as coatings and multilayer designs, or matrix systems with low moisture uptake and stable interphases. Humidity stability should be treated as a mandatory qualification metric rather than a secondary characterization.

Electrical stressing introduces an additional degradation pathway through localized Joule heating. In percolated networks, current concentrates at high resistance tunneling junctions, generating local temperature rises that soften the polymer and damage interfaces. These hotspots can nucleate micro-voids and disrupt network continuity, particularly in thin films and printed electronics [167,168]. Networks operating close to percolation are especially vulnerable. Electrical reliability therefore requires architectures with reduced junction resistance, improved thermal dissipation, and distributed conduction pathways, often achieved through hybrid fillers or continuous frameworks. A persistent limitation in the literature is the lack of standardized, quantitative reliability characterization. SEM imaging provides localized dispersion information but cannot capture network continuity at device-relevant length scales. Reproducible multifunctional performance requires quantitative quality-control descriptors, including electrical uniformity mapping, rheological indicators of network formation, and filler integrity metrics such as CNT length distribution and graphene defect density [169]. Processing history must also be reported, as shear conditions, mixing sequence, and cure schedule directly determine network topology and interface state. Without such metrics, property scatters cannot be mechanistically interpreted. Qualification protocols must be application-specific but mechanistically grounded. Sensors require resistance drift and gauge factor retention under cyclic strain and temperature variation. EMI shielding materials must demonstrate shielding effectiveness retention after bending and environmental aging. Thermal management components should be evaluated using assembled thermal resistance stability under thermal cycling rather than bulk conductivity alone [170]. Across applications, failure analysis should explicitly correlate performance degradation with microstructural damage metrics such as microcrack density, debonding fraction, and network disconnection.

As summarized in Table 8, durability in carbon/polymer composites is fundamentally a junction- and interface-evolution problem. Cyclic strain primarily enlarges tunneling gaps and disconnects pathways, leading to baseline resistance drift and hysteresis growth. Thermal cycling accelerates debonding and microcracking through thermal expansion mismatch, increasing junction and thermal boundary resistance. Humidity and electrical stressing introduce additional degradation through swelling-induced gap growth and hotspot-driven micro-void formation. Consequently, robust multifunctional performance requires junction redundancy through hybrid or 3D architectures, suppression of dispersion defects, and qualification using retention metrics such as conductivity, shielding effectiveness, strain response, and thermal resistance under realistic cycling and aging protocols.

Under realistic service conditions, the conductive network in carbon/polymer composites evolves due to coupled mechanical, thermal, and environmental effects that alter tunneling gaps and junction integrity. Under cyclic mechanical loading, progressive separation of conductive fillers leads to an increase in tunneling distance that can be described by a phenomenological relation, Δs(N) = A(1 − e^(−bN)) + cN, where Δs is the increase in interparticle gap after N cycles and the parameters A, b, and c represent rapid initial rearrangement and long-term creep-driven separation [170,171,172]. This results in an initial rapid increase in resistance followed by gradual drift, with typical CNT/elastomer systems exhibiting baseline resistance changes of ~15–30% after 10^3^ cycles. Environmental humidity introduces an additional degradation mechanism through moisture-induced swelling of the polymer matrix, which increases the effective tunneling gap according to Δs_hum ≈ s_0_·α_s·M_t/3, where M_t is the moisture uptake [173]. For example, ~2 wt% moisture absorption in polar matrices such as polyamide can increase the tunneling gap by ~0.15–0.25 nm, leading to a 2–5× increase in junction resistance due to the exponential dependence of tunneling conduction on gap distance. Thermal cycling further accelerates degradation through thermal expansion mismatch between fillers and matrix, generating interfacial stresses that promote debonding and microcrack formation [174]. Collectively, these mechanisms demonstrate that durability in carbon/polymer composites is governed by progressive evolution of tunneling gaps, junction resistance, and interfacial integrity, rather than bulk material degradation alone.

## 8. Sustainability, Circular Manufacturing and Outlook

Sustainability has become a critical constraint for the industrial translation of carbon/polymer composites, particularly in high-volume sectors such as automotive, consumer electronics, and construction. Conventional high-performance systems often rely on fossil-derived thermosets with limited recyclability, while carbon nanofillers introduce additional challenges related to separation, dispersion stability, and environmental persistence [81,176,177]. As a result, current research has shifted from maximizing peak properties toward designing composites that retain multifunctionality over multiple life cycles, enable responsible end-of-life management, and reduce processing energy and solvent intensity. Achieving these objectives requires sustainability to be embedded within the microstructure design framework rather than addressed post hoc.

**Figure 11:**
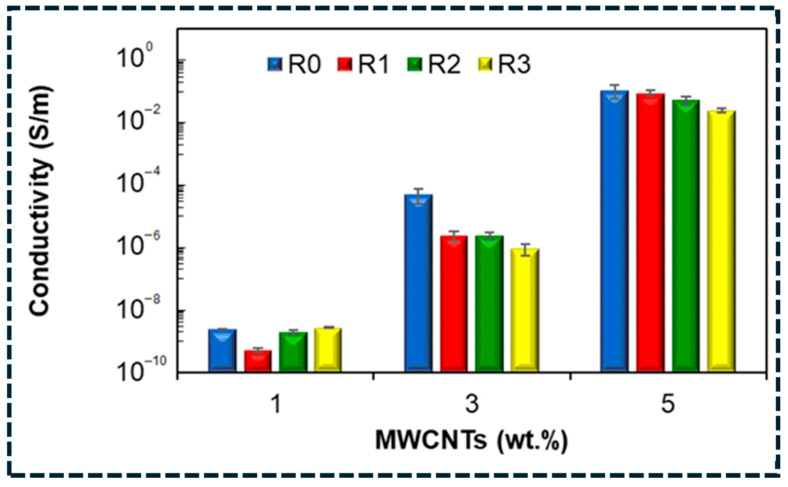
Electrical conductivity retention of MWCNT/polymer composites after successive recycling cycles (R0–R3) at 1, 3, and 5 wt% CNT, showing progressive conductivity loss due to percolated network degradation [183].

A primary sustainability limitation is the restricted recyclability of many carbon/polymer systems [178,179,180]. Thermosets offer strong interfacial coupling and network stability but cannot be reprocessed due to their crosslinked structure, restricting circular pathways [181]. Thermoplastics are intrinsically recyclable; however, repeated thermal–shear histories during reprocessing can degrade conductive networks through CNT shortening, graphene defect evolution, and filler reaggregation, compromising percolation stability [182,183]. Figure 11 illustrates this effect, showing a progressive decrease in electrical conductivity from R0 to R3 at constant MWCNT loading, consistent with network fragmentation and junction rearrangement during recycling. These observations demonstrate that recyclability must be evaluated using conductivity retention rather than polymer reprocessability alone [183].

**Figure 12:**
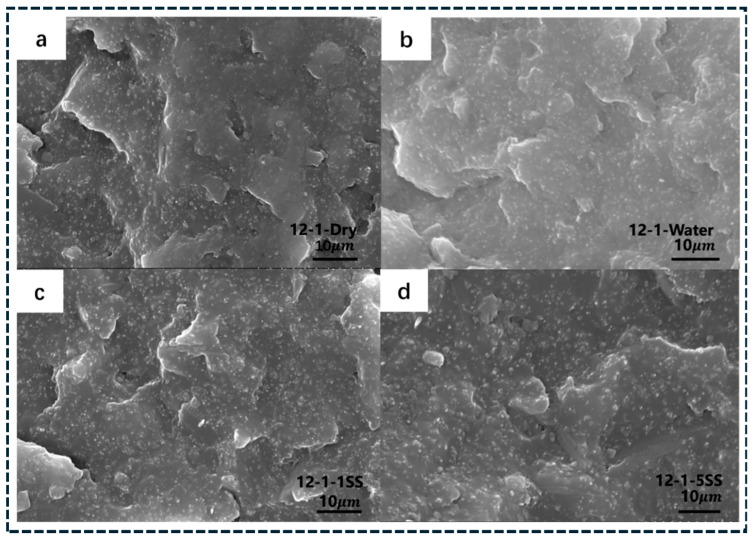
SEM micrographs of conductive composites after environmental aging: (**a**) dry, (**b**) water immersion, (**c**) simulated sweat (1SS), and (**d**) concentrated simulated sweat (5SS). The absence of major microcracks or network collapse indicates aging is dominated by junction and interface modifications rather than catastrophic structural damage [184].

Environmental durability further constrains sustainable deployment. Figure 12a–d shows that exposure to humid and sweat environments does not induce severe microcracking or macroscopic network collapse, indicating that degradation is governed primarily by gradual interface and junction evolution rather than catastrophic morphological failure [184]. Such subtle changes are sufficient to degrade electrical transport, EMI shielding, and sensing performance, reinforcing the need for σ retention and junction stability metrics when assessing long-term reliability. Overall, sustainable carbon/polymer composites must be designed to preserve filler integrity, junction resistance, and interphase stability under repeated thermal, mechanical, and environmental cycling, rather than focusing solely on matrix recyclability.

Bio-based and biodegradable matrices such as PLA, PHA, and starch-based blends offer reduced carbon footprint and improved circularity, but their moisture sensitivity and limited thermal stability impose additional microstructural constraints [185,186]. Matrix swelling and plasticization increase tunneling gaps and reduce conductivity, while hydrolysis weakens interfacial adhesion and accelerates crack initiation. As a result, sustainable bio-based composites require network architectures that are intrinsically tolerant to junction instability. Hybrid networks with junction redundancy, segregated conductive pathways, and continuous frameworks are particularly effective because they preserve connectivity even when local tunneling gaps increase [185]. Barrier coatings and low-permeability multilayer designs further mitigate moisture-driven degradation. Thus, the sustainability advantage of bio-based matrices can only be realized when humidity stability is explicitly engineered into the network design. An additional sustainability pathway is the use of bio-derived or waste-derived carbon fillers, including lignin-based fibers, biomass-derived activated carbon, pyrolyzed cellulose carbon, and reclaimed industrial carbon [187,188]. While these fillers reduce energy and environmental cost, they often exhibit higher defect density, broader size distributions, and variable surface chemistry, which increase percolation variability and junction resistance scatter [188]. Consequently, dispersion-tolerant architectures and standardized characterization of surface area, defect density, and conductivity are essential to ensure reproducible performance when using sustainable carbon sources.

Recyclable thermosets, particularly vitrimers and dynamic covalent networks, offer a promising compromise between mechanical performance and circularity. Dynamic bond exchange enables reshaping and repair and may partially restore conductive pathways through microstructural rearrangement [189]. However, repeated reprocessing can alter filler spacing and interphase chemistry, leading to gradual conductivity drift. Design rules for vitrimer–carbon composites must therefore prioritize percolation stability and property retention over multiple recycling cycles. From a manufacturing perspective, sustainability favors low-solvent and low-energy routes. Although solution processing provides excellent dispersion, its environmental burden motivates a shift toward melt-based, latex-based, and reactive processing. Additive manufacturing further reduces waste through near-net-shape fabrication and enables functionally graded architectures that minimize filler usage [190]. However, printable inks and filaments must maintain stable dispersion and filler integrity without relying on high solvent content. Integrating life-cycle assessment with microstructure design can identify architectures that maximize functional performance per unit environmental impact.

Looking ahead, several priorities will define progress in sustainable carbon/polymer composites. Predictive models linking processing rheology, dispersion evolution, and percolation transport are required to improve reproducibility. Interface engineering should transition from irreversible functionalization toward dynamic, transport-preserving chemistry [191]. Architected networks produced by segregated structures, 3D frameworks, and additive manufacturing must be scaled with standardized protocols. Finally, durability and sustainability must be co-optimized by designing networks that retain conductivity and shielding under cyclic strain, thermal aging, and humidity while remaining recyclable or repairable. Therefore, sustainability is reshaping carbon/polymer composite design from peak property optimization toward life-cycle stability. Retention of filler integrity, junction resistance, and interphase stability is critical for recycled, bio-based, and dynamically reprocessable systems [192]. Future advances will rely on predictive modeling, standardized durability metrics, and circular manufacturing strategies that embed sustainability directly into microstructure design. Table 9 illustrates that sustainability constraints map directly onto microstructural stability challenges, emphasizing that functional composites must be evaluated by property retention and uniformity, rather than initial performance alone.

## 9. Conclusions

Carbon/polymer composites have evolved from conventional conductive plastics into microstructure-engineered multifunctional systems, where mechanical, electrical, thermal, and electromagnetic properties are governed primarily by network formation rather than filler loading alone. Across 0D–3D carbon architectures, performance is dictated by the coupled effects of dispersion, interphase characteristics, and network topology under processing-induced shear and thermal histories. Electrical behavior is controlled by percolation and tunneling-dominated junctions, leading to nonlinear conductivity and high strain sensitivity near the percolation threshold, while thermal transport is often limited by Kapitza and junction resistances. Achieving reproducible multifunctionality therefore requires architecture-driven strategies that enhance connectivity, reduce junction losses, and stabilize conductive pathways. CNT and CNF networks provide low percolation thresholds and strong piezoresistive responses but are sensitive to dispersion and processing damage. Graphene-based systems offer planar conduction but are limited by restacking and junction resistance, whereas carbon black ensures robustness at higher loadings. Hybrid architectures enable true synergy through engineered network topology, such as CNT bridging and hierarchical densification, while three-dimensional carbon frameworks provide continuous pathways for improved conductivity and EMI shielding at low filler content. Matrix selection and segmental mobility govern tunneling gap stability and durability, while processing routes such as extrusion and additive manufacturing define network alignment and continuity. Long-term performance is limited by network evolution under cyclic strain, thermal cycling, and humidity, leading to resistance drift and conductivity loss. Therefore, standardized quantitative characterization and sustainable design strategies, including recyclable matrices and dynamic networks, are essential. Future advances will rely on predictive modeling, interface engineering, and scalable architected networks to achieve durable, consistent, and multifunctional performance.

From an application-driven perspective, the best-performing carbon/polymer composites are those that achieve optimized network topology and junction engineering rather than simply high filler loading. Hybrid architectures, particularly CNT–graphene and CNT–carbon black systems, consistently demonstrate superior multifunctional performance due to the reduced percolation threshold, increased junction density, and improved connectivity stability under mechanical and environmental loading. Three-dimensional continuous carbon frameworks, such as foams and aerogels, represent the most effective strategy for achieving high electrical and thermal conductivity at low filler fractions by minimizing junction resistance and enabling continuous transport pathways. For sensing applications, CNT/elastomer composites exhibit the highest piezoresistive sensitivity due to tunneling-dominated transport near the percolation threshold, while hybrid systems improve signal stability and durability. In EMI shielding, hybrid and 3D carbon architectures provide the most efficient performance by combining high conductivity with enhanced absorption through multiple internal reflections. For thermal management, aligned graphene networks and vertically aligned CNT frameworks offer the highest effective thermal conductivity by reducing Kapitza resistance effects and enabling directional heat transport. Overall, the most effective carbon/polymer composites are those that integrate hybrid or hierarchical architectures with controlled dispersion and interface engineering to achieve stable, scalable, and application-specific multifunctional performance.

The selection of the polymer matrix plays a critical role in determining the dispersion quality, interfacial interactions, and long-term stability of carbon/polymer composites. Thermoplastics such as polypropylene (PP), polyethylene (PE), polycarbonate (PC), and polyamide (PA) are well suited for large-scale processing due to their melt processability and recyclability; however, they often require compatibilization strategies to achieve uniform filler dispersion and stable conductive networks. Thermosetting polymers, including epoxy and vinyl ester resins, provide strong interfacial bonding and structural rigidity, enabling stable network formation and superior mechanical reinforcement, making them particularly suitable for structural and EMI shielding applications. In contrast, elastomeric matrices such as polydimethylsiloxane (PDMS), thermoplastic polyurethane (TPU), and rubber systems offer high deformability and enable large strain-induced tunneling variations, resulting in exceptional piezoresistive sensitivity for sensing applications, albeit with increased hysteresis and long-term drift. Overall, thermoplastics are preferred for scalable and industrial applications, thermosets for structural and stable multifunctional performance, and elastomers for flexible and high-sensitivity sensing systems. This comparison highlights that matrix selection must be aligned with the targeted balance between processability, transport performance, and durability.

From an application-specific perspective, the most promising carbon/polymer composites can be identified based on their underlying transport mechanisms and network architecture. For flexible and wearable sensing applications, CNT/elastomer composites (e.g., CNT/PDMS, CNT/TPU) exhibit the highest piezoresistive sensitivity due to tunneling-dominated conduction near the percolation threshold, while hybrid systems incorporating secondary fillers such as carbon black or graphene improve signal stability and reduce hysteresis through enhanced junction density. For electromagnetic interference (EMI) shielding, hybrid CNT–graphene composites and three-dimensional (3D) carbon architectures represent the most effective systems, as they combine high electrical conductivity with enhanced electromagnetic wave absorption through multiple internal reflections and improved network connectivity at low filler loading. In thermal management applications, aligned graphene networks, vertically aligned CNT frameworks, and continuous 3D carbon structures provide the highest effective thermal conductivity by minimizing Kapitza resistance and establishing continuous heat conduction pathways. These observations demonstrate that the most promising composites are those that integrate controlled network topology, optimized junction characteristics, and application-specific architecture to achieve high performance with stability and scalability.

**Design Rule**

Based on the mechanistic framework developed in this review, a set of application-independent, structure–processing–property design rules is proposed to guide the development of carbon/polymer composites with reproducible multifunctional performance:

DR-1: Target high effective aspect ratio (AR_eff > 100) for 1D fillers such as CNT/CNF to achieve low percolation thresholds (φc < 1 vol%). Aspect ratio directly governs network connectivity and percolation efficiency.

DR-2: Control dispersion and agglomeration to minimize variability in network topology. Uniform dispersion reduces local defects and ensures consistent electrical and mechanical performance.

DR-3: Minimize the mean tunneling gap (s < 1.5 nm) through controlled processing and interface design, as tunneling resistance depends exponentially on interparticle separation.

DR-4: Increase junction density and connectivity using hybrid filler architectures (e.g., CNT–graphene or CNT–carbon black), where secondary fillers densify networks and reduce reliance on high-resistance tunneling pathways.

DR-5: Optimize the polymer matrix glass transition temperature (Tg) such that Tg is at least 30–40 K higher than the service temperature to stabilize tunneling gaps and suppress conductivity drift.

DR-6: Balance interfacial adhesion and transport efficiency. Strong interfaces improve load transfer but may increase electrical and thermal resistance due to interphase formation.

DR-7: For thermal management applications, reduce Kapitza resistance and junction bottlenecks by employing aligned fillers or continuous 3D carbon frameworks instead of randomly dispersed networks.

DR-8: Utilize processing routes (e.g., extrusion, injection molding, additive manufacturing) as microstructure-control tools to tailor filler alignment, anisotropy, and network continuity.

DR-9: Design networks with redundancy (e.g., hybrid or 3D architectures) to maintain connectivity under cyclic mechanical loading and minimize durability-related degradation.

DR-10: For sensing applications, operate near but above the percolation threshold (φ/φc ≈ 1.1–1.5) to balance sensitivity and signal stability.

DR-11: Evaluate durability using stability metrics such as conductivity retention, resistance drift, and performance under cyclic strain, thermal aging, and humidity exposure rather than relying on initial property values.

DR-12: Quantify microstructure using standardized metrics such as connectivity index, agglomerate size distribution, and conductivity mapping to ensure reproducibility and enable structure–property correlation.

These design rules provide a unified framework linking dispersion, network topology, junction physics, and interface engineering to achieve reliable electrical, thermal, and mechanical performance in carbon/polymer composites.

## Figures and Tables

**Table 1:** Comparison of the present review with existing reviews on carbon/polymer composites.

Review Focus	Key Gap	Unique Contribution of This Review
Scope of carbon fillers [1,6,32]	No unified view across 0D–3D fillers	Unified 0D/1D/2D/3D comparison based on governing mechanisms
Treatment of microstructure [5,6]	No quantitative link to percolation/anisotropy	Dispersion and topology are treated as core, quantifiable variables
Electrical transport mechanism [31]	Tunneling, junctions not integrated	Percolation–tunneling framework with strain/thermal stability
Thermal transport [32]	Kapitza and junction limits ignored	Interface- and junction-limited heat transport framework
Interface engineering [36]	Transport penalties overlooked	Explicit adhesion–transport trade-off and dynamic interfaces
Hybrid filler systems [31]	Mechanism unclears	Mechanistic hybrid design rules for low pc and stability
3D architectures [6]	No transport rationale	3D networks as percolation-bypass architectures
Manufacturing emphasis [5]	Limited scalability guidance	Scalable processing with reproducibility metrics
Additive manufacturing [3]	No network/anisotropy design	AM as microstructure-programming tool
Reliability & durability [31]	Aging and fatigue neglected	Network degradation mechanisms and reliability roadmap
Sustainability [9]	Not design-integrated	Circularity-driven microstructure design
Design outcome [31]	No predictive rules	Application-specific, mechanism-based design rules

**Table 2:** Mechanistic comparison of carbon architectures in polymer composites.

Architecture	Key Descriptor	Reinforcement	Transport Mechanism	Limitation/Design Role
0D (carbon black) [71]	Aggregate connectivity	Hydrodynamic stiffening; polymer confinement	Percolation through aggregate contacts	High loading required; viscosity increase; ductility reduction; effective as secondary network densifier
1D (CNT/CNF) [72]	Effective aspect ratio; length retention; bundle size	Shear-lag load transfer; crack bridging/pull-out	Percolation with tunneling-dominated junctions	Agglomeration and shear-shortening increase percolation threshold; causes property scatter; best percolating backbone
2D (graphene/GNP) [73]	Platelet junction density; alignment factor	Crack deflection; load redistribution	Junction-limited transport; anisotropic conduction	Restacking and contact resistance limit network continuity; suitable for aligned heat/EMI pathways
2D (GO/rGO) [74]	Defect density; functional group coverage	Strong interfacial anchoring	Defect-limited conduction with percolation	Dispersion–conductivity trade-off; best for barrier or mechanically reinforced systems
Hybrid (CNT–graphene/CNT–CB) [75]	Bridging probability; junction redundancy	Network-mediated stress redistribution	Lower percolation threshold; reduced junction losses	Sensitive to mixing sequence and segregation; enables low loading multifunctionality and stability
3D continuous framework [76]	Skeleton continuity; infiltration completeness	Framework bridging; crack deflection	Continuous conduction (percolation bypass)	Infiltration defects and thermal boundary resistance; best for EMI/thermal at low loading

**Table 4:** Processing routes as microstructure generators for carbon/polymer composites.

Processing	Microstructure Control	Anisotropy Outcome	Defect	Suitability
Melt compounding (extrusion) [93]	Shear history; viscosity; residence time	Dispersion with partial alignment; percolation set by effective aspect ratio	CNT shortening; graphene restacking; agglomerates	Thermoplastic CNT/GNP and hybrid composites
Masterbatch dilution [94]	Concentrate quality; dilution sequence	Improved uniformity with retained backbone	Persistent agglomerates	CNT-based and hybrid systems
Injection molding [95]	Flow field; cooling rate	Skin–core alignment; high anisotropy	Reduced through-thickness transport; voids	Directional conduction and EMI components
Solution blending/casting [96]	Solvent stabilization; evaporation rate	Low percolation networks in films	Drying-induced reaggregation	Coatings and thin films
In situ polymerization/cure [97]	Pre-gel mixing; gelation timing	Network locked at gel; shrinkage-modified gaps	Residual stress; microcracking	Thermoset CNT/graphene composites
Additive manufacturing (FDM/DIW) [98]	Nozzle shear; print path	Programmable anisotropy; voxel-level percolation	Interlayer resistance; porosity	Architected sensors, EMI and thermal components

**Table 5:** Studies linking network topology and junction physics to electrical, thermal, and mechanical responses in carbon/polymer composites.

Reference	System	Core Mechanistic Contribution	Reported Outcome
Casto et al. [115]	CNT/fluid interface	Kapitza (thermal boundary) resistance governs heat transfer	Interfacial resistance limits thermal transport
Noh et al. [116]	Epoxy/CNT/graphene	Hybrid networks reduce junction resistance and improve connectivity	Lower percolation threshold; enhanced conductivity
Bao et al. [117]	CNT composites (modeling)	Dispersion, alignment, and agglomeration control transport	Conductivity anisotropy predicted by network arrangement
Haghgoo et al. [118]	CF/CNT hybrid	Network density and CNT aspect ratio govern percolation	Percolation shift and conductivity enhancement
Hu et al. [119]	SWCNT networks	Stick percolation in CNT networks	Percolation behavior depends on network density
Alexeev et al. [120]	Graphene interfaces	Coupling strength controls Kapitza resistance	Interface-dominated thermal transport
Lee et al. [121]	CNT/polymer	Tunneling resistance at CNT junctions	Conductivity governed by tunneling gap distribution
Florencia et al. [122]	CNT networks	Voltage-dependent tunneling conduction	Nonlinear electrical response
Zare et al. [123]	CNT composites	Interphase thickness controls tunneling distance	Reduced percolation threshold; conductivity increase
Kirkpatrick et al. [124]	Percolation theory	Critical scaling of conductivity	Universal percolation behavior
Last et al. [125]	Percolation transition	Conductivity transition at percolation	Sharp transport transition
Balberg et al. [126]	Rod networks	Aspect ratio controls percolation threshold	Lower pc with higher aspect ratio
Bauhofer et al. [127]	CNT/polymer	Dispersion and junction resistance dominate transport	Wide conductivity scatter linked to processing
Celzard et al. [128]	Carbon-filled polymers	Tunneling–percolation conduction	Conductivity scaling depends on aggregation
Terrado et al. [129]	CNT composites	Dispersion stability controls conductivity	Improved dispersion enhances conductivity
Battiston et al. [130]	CNT/polymer	Tunneling gap modulation under strain	Piezoresistive response
Davidson et al. [131]	CNT/epoxy	Agglomeration limits reinforcement	Modulus increase; strength defect-limited
Delgado et al. [132]	CNT/polymer	Effective aspect ratio governs reinforcement	Modulus enhancement depends on dispersion
Liu et al. [133]	Thermal transport model	Interface resistance limits effective k	Interface-controlled thermal conductivity
Arulkumaran et al. [134]	CNT thermal transport	Phonon scattering and interface resistance dominate	Limited thermal enhancement
Kietzke et al. [135]	CNT interfaces	Thermal boundary resistance dominates heat flow	Interface-limited heat transfer
Li et al. [136]	GNP/polymer	Junction-controlled conduction and alignment	Anisotropic conductivity
Jnag et al. [137]	CNT–graphene hybrid	Bridging reduces junction resistance	Lower percolation; stable conductivity
Falco et al. [138]	Graphene/polymer	Dispersion quality governs transport scatter	Conductivity variability quantified
Beaucage et al. [139]	Carbon black/polymer	Aggregate morphology controls percolation	Percolation sensitive to aggregate structure
Chen et al. [140]	CNT/polymer	Improved dispersion enhances network connectivity	Reduced percolation threshold; conductivity increase

**Table 6:** Unified microstructural descriptors linking network topology and interface physics to electrical, thermal, and mechanical responses in carbon/polymer composites.

Microstructure Descriptor	Practical Quantification	Electrical Response	Thermal Response	Mechanical Response
Network continuity (CI) [151]	3D tomography, conductivity mapping, graph reconstruction	Lower percolation threshold; higher and more stable conductivity	Fewer transport bottlenecks; improved effective thermal conductivity	Improved stress redistribution: defect sensitivity governs brittleness
Junction/contact density (Nc) [152]	Contact counting, graph models, rheological plateau modulus	Higher conductivity with reduced scatter; lower piezoresistive sensitivity	Increased junction contribution unless continuous pathways form	Increased stiffness, agglomerated contacts act as stress concentrators
Mean tunneling gap (⟨s⟩) and distribution P(s) [153]	Piezoresistive calibration, tunneling models	Smaller gaps increase conductivity; near-percolation gaps enhance sensitivity but increase drift	Minor influence due to phonon-dominated transport	Large gaps weaken junctions and reduce fatigue resistance
Junction resistance distribution (Rj) [154]	Local I–V mapping, resistor-network fitting	Lower and narrower resistance improves conductivity reproducibility	Reduced temperature drop at contacts improves heat transport	Stronger junctions improve strength but may reduce ductility
Interphase thickness [155]	DSC, DMA, spectroscopy, interphase modeling	Reduced percolation threshold but potential tunneling penalty	Increased thermal boundary resistance unless coupling improves	Improved load transfer up to brittleness limit
Kapitza resistance (Rk) [156]	TDTR, FDTR, fitted thermal models	Indirect electrical effect via interface chemistry	Primary limiter of effective thermal conductivity	Improved interface stability reduces debonding
Effective aspect ratio (AR_eff) [157]	CNT length statistics, Raman defect analysis, SEM	Lower percolation threshold and improved network stability	Beneficial only for aligned or continuous pathways	Enhanced shear-lag load transfer; shortening degrades reinforcement
Dispersion defect index (DI) [158]	Agglomerate statistics, void fraction analysis	Increased conductivity scatter and unstable percolation	Elevated contact resistance and porosity reduce heat transport	Dominant crack initiation sites; reduced strength and toughness

**Table 7:** Application-driven design rules for carbon/polymer composites (mechanism-based).

Application	Key Metric	Governing Mechanism	Preferred Architecture	Primary Degradation Mode	Reliability Metric
Flexible sensors (strain/pressure) [159]	Gauge factor, ΔR/R	Tunneling-dominated conduction near percolation	CNT/elastomer; CNT–CB or CNT–graphene hybrids; segregated networks	Viscoelastic drift; microcrack-driven network rupture	ΔR/R retention under cyclic loading; baseline drift
EMI shielding (rigid/flexible) [160]	Shielding effectiveness (SE)	Network continuity and absorption losses	CNT–graphene hybrids; layered or 3D networks	Conductivity loss under strain; delamination	SE retention after bending and aging
Thermal interface materials (TIMs) [161]	Through-plane thermal resistance	Junction-limited heat transfer; Kapitza resistance	Vertically aligned CNT/graphene; 3D frameworks	Thermal cycling-induced debonding	Thermal resistance stability after cycling
Heat spreaders/casings [162]	In-plane thermal conductivity	Alignment-controlled anisotropic transport	Aligned graphene platelets; 1D–2D hybrids	Warpage; anisotropic cracking	Conductivity retention after thermal aging
Conductive/antistatic parts [163]	Electrical resistivity	Stable percolation above threshold	CNT thermoplastics; CNT–CB hybrids	Crack- and humidity-induced resistivity drift	Resistivity stability vs temperature and humidity
Energy devices (electrodes/electrolytes) [164]	Electronic transport; cycling stability	Percolated electronic networks with interface control	Porous 3D CNT/graphene frameworks	Interface degradation; pore blockage	Conductivity and capacity retention during cycling

**Table 8:** Durability failure mechanisms and qualification metrics for carbon/polymer composite.

Service Condition	Key Degradation Mechanism	Main Property Affected	Typical Drift	Mitigation Strategy	Qualification Metric
Cyclic strain [169]	Tunneling gap growth; junction loss	σ, GF	R_0_ drift; hysteresis ↑	Hybrid networks; defect control	σ or ΔR/R retention (10^3^–10^6^ cycles)
Thermal aging/cycling [170]	CTE mismatch; debonding; microcracks	Rth, σ, SE	Rth ↑; σ ↓	Aligned/continuous paths; high Tg	ΔRth after cycling; σ retention
Humidity exposure [171]	Swelling; plasticization; interface weakening	Resistivity	Baseline drift	Barriers; low-moisture matrices	Resistivity drift vs RH
Electrical stress [172]	Hotspots at junctions; voiding	σ	Sudden σ drop	Junction densification; heat paths	σ retention under current cycling
UV/oxidative [173]	Chain scission; embrittlement	Mechanical, SE	Crack density ↑	UV stabilizers; coatings	SE retention after UV aging
Creep/static load [174]	Polymer relaxation; network shift	σ stability	Log-time drift	High Tg; network redundancy	Resistance drift under hold
Reproducibility [175]	Dispersion non-uniformity	All properties	Property scatter	Process control; metrics	σ mapping; G′ plateau

Note: R_0_ drift = baseline resistance changes with time/cycles, ΔR/R decay = sensor signal becomes weaker after cycling, Hysteresis ↑ = loading and unloading curves do not overlap (bigger loop), σ ↓ = conductivity decreases after aging/strain, Rth ↑ = thermal resistance increases after thermal cycling, SE drop = EMI shielding effectiveness decreases after bending/aging.

**Table 9:** Sustainability-driven material and processing strategies for carbon/polymer composites.

Sustainability Requirement	Key Challenge	Governing Microstructural Mechanism	Practical Strategy	Main Performance Risk	Recommended Metric
Recyclability (thermoplastics) [189]	Network degradation during reprocessing	CNT shortening, graphene restacking, tunneling gap increase	Masterbatch approach, low-shear reprocessing, hybrid or segregated networks	Conductivity and sensing drift	Conductivity retention after 3–5 cycles; percolation threshold shift
Reprocessable thermoset [181]	Conventional thermosets are non-recyclable	Permanent crosslinked network	Vitrimers, dynamic covalent matrices, reversible interfaces	Conductivity or shielding drift after reprocessing	Property retention after reprocessing; σ/SE stability
Bio-based matrices [190]	Moisture sensitivity and swelling	Increased tunneling distance, interphase softening	Barrier coatings, low-permeability blends, junction-redundant networks	Resistivity increase, calibration drift	Conductivity drift versus humidity; aging stability
Bio-/waste-derived carbons [191]	Variability in filler quality	Junction resistance scatter, percolation variability	Standardized filler characterization, dispersion-tolerant hybrids	Batch-to-batch property scatter	Dispersion index and conductivity uniformity mapping
Solvent-free manufacturing [192]	Dispersion loss without solvents	Drying-induced aggregation, limited deagglomeration	Melt, latex, or reactive processing	Higher percolation threshold	Energy intensity; conductivity versus loading reproducibility
Material efficiency [193]	High filler loading increases cost and brittleness	Viscosity rise, defect-driven fracture	3D frameworks, aligned or segregated networks, AM patterning	Toughness loss, processing difficulty	Functional performance per filler fraction

## Data Availability

The data supporting the reported results is not stored in any publicly archived datasets. The readers can contact the corresponding author for any further clarification of the results obtained.

## Nomenclature: Acronyms and Abbreviations

AM	additive manufacturing
AR	aspect ratio
AR_eff	effective aspect ratio
CB	carbon black
CF	carbon fiber
CI	connectivity index (network continuity metric)
CNF	carbon nanofiber
CNT	carbon nanotube
CTE	coefficient of thermal expansion
DI	dispersion defect index/agglomerate fraction
DMA	dynamic mechanical analysis
DSC	differential scanning calorimetry
DIW	direct ink writing
EMI	electromagnetic interference
FDM	fused deposition modeling
GF	gauge factor
GNP	graphene nanoplatelet
GO	graphene oxide
IFSS	interfacial shear strength
PLA	polylactic acid
PHA	polyhydroxyalkanoate
RVE	representative volume element
rGO	reduced graphene oxide
RH	relative humidity
SE	shielding effectiveness
SEM	scanning electron microscopy
TBR	thermal boundary resistance
TDTR/FDTR	time-/frequency-domain thermoreflectance
TIM	thermal interface material
Tg	glass transition temperature
UTS	ultimate tensile strength
**General/microstructure descriptors**	
φ	filler volume fraction
φc (pc)	percolation threshold/critical filler fraction
CI	connectivity index/network continuity metric (–)
Nc	junction/contact density (contacts per unit volume or per filler)
DI	dispersion defect index/agglomerate fraction
s, ⟨s⟩	tunneling gap distance and mean tunneling gap (nm)
P(s)	probability distribution of tunneling gap distances
**Electrical transport**	
σ	electrical conductivity (S·m^−1^)
σ0	conductivity prefactor in percolation law (S·m^−1^)
ρ	electrical resistivity (Ω·m)
t	percolation critical exponent
R0	tunneling resistance prefactor (Ω)
Rc	contact (ohmic) junction resistance (Ω)
Rt	tunneling junction resistance (Ω)
β	tunneling decay coefficient (nm^−1^)
R0 drift	baseline resistance drift during cycling/aging
ΔR/R0	normalized resistance change (sensor response)
**Thermal transport**	
keff (k_eff)	effective thermal conductivity of composite (W·m^−1^·K^−1^)
km	thermal conductivity of polymer matrix (W·m^−1^·K^−1^)
kf	thermal conductivity of filler (W·m^−1^·K^−1^)
Rk	Kapitza (thermal boundary) resistance at filler–matrix interface (m^2^·K·W^−1^)
Gk	thermal boundary conductance (Gk = 1/Rk) (W·m^−2^·K^−1^)
Rj	filler–filler thermal contact resistance (K·W^−1^ or m^2^·K·W^−1^; definition dependent)
Rth	total thermal resistance (assembly/device) (K·W^−1^)
ΔRth	change in thermal resistance after cycling/aging (K·W^−1^)
q	heat flux (W·m^−2^)
ΔT	temperature drop across an interface or junction (K)
**Mechanical reinforcement**	
E	elastic modulus (GPa)
ε	strain
τi	interfacial shear strength (MPa)
L	filler length (μm)
lc	critical length for load transfer (μm)
d	filler diameter (nm)
σf	filler tensile strength (GPa)
**Application-level outputs**	
SE (dB)	electromagnetic interference shielding effectiveness (dB)
k	thermal conductivity used in application metrics (W·m^−1^·K^−1^)
EMI	electromagnetic interference
TIM	thermal interface material
